# Gut microbiome is associated with stereotypic behavior of the giant panda (*Ailuropoda melanoleuca*)

**DOI:** 10.1371/journal.pone.0357625

**Published:** 2026-09-23

**Authors:** Anqi Hu, Jinyuan Liu, Peng Chen, Kuixing Yang, Fan Xu, Gai Luo, Jiali Zhang, Mengnan He

**Affiliations:** 1 The Conservation of Endangered Wildlife Key Laboratory of Sichuan Province, Chengdu Research Base of Giant Panda Breeding, Chengdu, Sichuan, China; 2 College of Veterinary Medicine, Jilin Agricultural University, Changchun, Jilin, China; 3 School of Pharmacy, Chengdu University, Chengdu, Sichuan, China; 4 School of Public Health, Chengdu Medical College, Chengdu, Sichuan, China; Sun Yat-Sen University, CHINA

## Abstract

The giant panda (*Ailuropoda melanoleuca*) is a relict species endemic to China. Captive breeding has boosted its population. However, some captive individuals exhibit stereotypic behavior. Though extensive research showing that host behavior can be influenced by gut microbiota via the gut-brain axis, the gut microbiota related to stereotypic behavior of giant pandas has not been reported. To explore gut microbial composition and functional characteristics of stereotypically behaving (SB) giant pandas, the behavior of 15 captive giant pandas was observed, and their feces were analyzed using 16S ribosomal RNA (rRNA) sequencing (n = 145) and metagenomic sequencing (n = 45). Our study revealed distinct gut microbial diversity, composition, and functions between SB giant pandas and normal controls, indicating gut microbial dysbiosis in SB giant pandas. *Serratia* sp. *Se-RSBMAAmG* was significantly enriched in SB giant pandas, five *Clostridium* species and *Lactococcus garvieae* exhibited upward trends in SB individuals, whereas the probiotic *Enterococcus hirae* showed the opposite trend compared with those in normal controls. The *Clostridium* bacteria may affect propionate metabolism and the gamma-aminobutyric acidergic (GABAergic) synapse pathway, potentially affecting the emergence of stereotypic behavior. These findings suggest a link between gut microbiota and stereotypic behavior of giant pandas, providing novel insights for their conservation.

## Introduction

The giant panda (*Ailuropoda melanoleuca*), an umbrella and flagship species, symbolizes global biodiversity [1]. The species encounters significant risks to its existence due to the destruction of its habitat by humans [2]. Currently, due to habitat protection and captive breeding, giant panda populations are showing a notable rise [3]. However, in the process of captive breeding, due to the influence of factors such as lack of stimulation, freedom restriction, and environmental stress in a captive environment, some giant pandas show stereotypic behavior, resulting in the degradation of their normal behavior and inability to breed, which seriously affects the conservation of giant pandas [4–6]. Stereotypic behavior is a repetitive, unvarying, and apparently functionless behavioral pattern typical of animals in some conditions of captivity [5]. Although environmental factors are known to influence its occurrence [7], the mechanism of stereotypic behavior in giant pandas is not clear. Notably, Stereotypic behavior is an iconic symptom of autism spectrum disorder (ASD) [8,9]. Extensive evidence has consistently confirmed that the gut microbiota influences host behavior via the gut-brain axis [10–12], a bidirectional link between gut and brain [13] that plays a key role in the development of ASD [14,15]. Currently, there is a substantial body of research on gut microbiota in ASD. Differences in the gut microbial composition between individuals with ASD and their controls were observed [16]. The abundance of specific bacterial genera in the gut was significantly higher in the ASD group than in the controls [17]. ASD with constipation is associated with changes in gut microbiota and an elevated level of propionate in the stool [18]. Research has shown that colonization by microbiota from ASD can effectively induce iconic autistic behaviors [14]. A link between the colonization of *Clostridium* bacteria in the gut and ASD was demonstrated [19]. Five new bacterial biomarkers for ASD prediction were identified [20]. *Turicibacter* and *Catenibacterium* were believed to be potential biomarkers for ASD [21]. However, the gut microbiota associated with the stereotypic behavior of giant pandas has not been reported.

**Fig 1:**
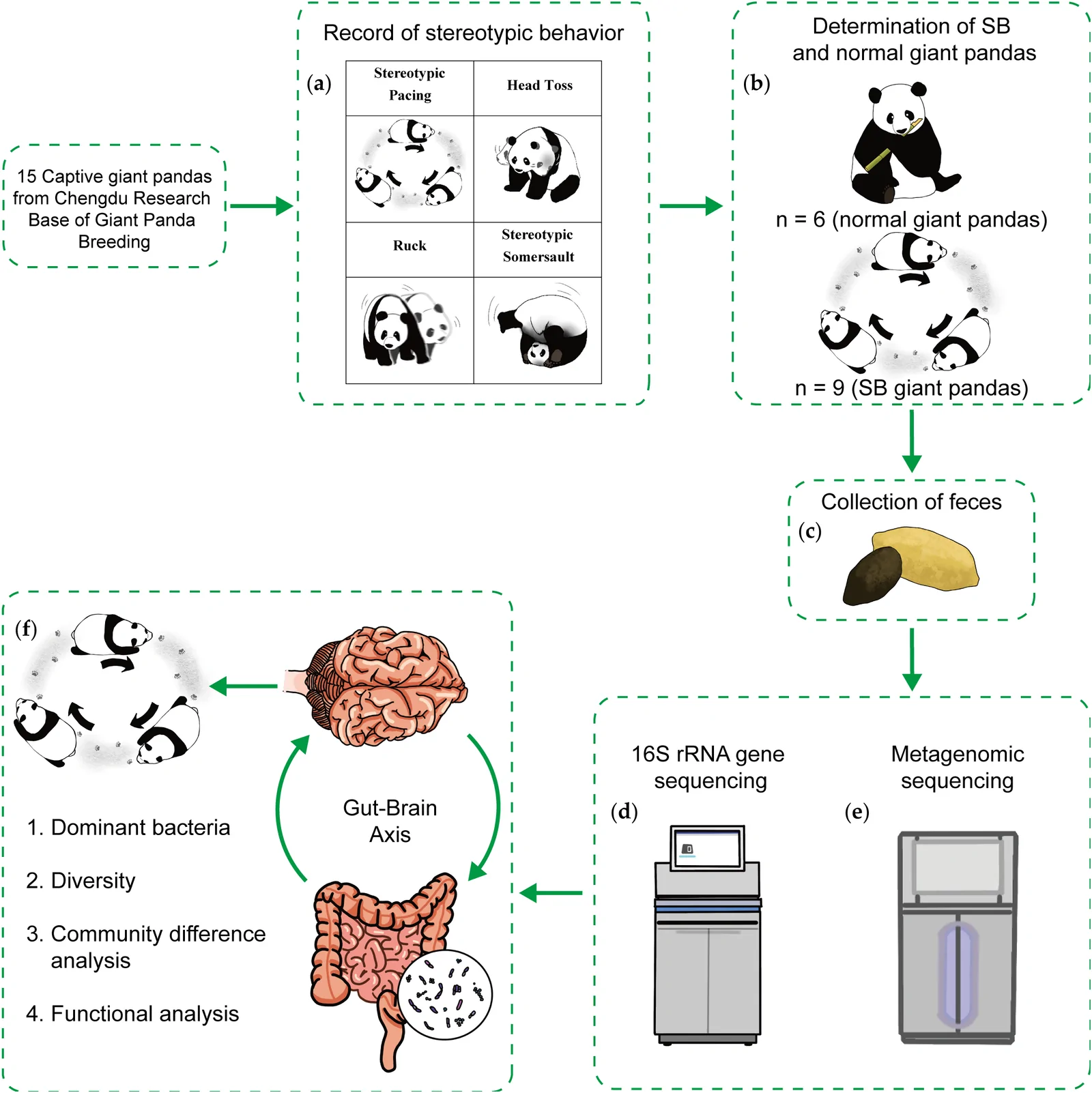
The general workflow diagram of the study. (**a**) Four major types of stereotypic behavior were observed, ranked by duration: stereotypic pacing, head toss, ruck, and stereotypic somersault. (**b**) Based on behavioral observation, nine giant pandas were selected as stereotypically behaving (SB) individuals, and six as normal controls. Fresh fecal samples were collected (**c**)and subjected to 16S ribosomal RNA (rRNA) gene sequencing (**d**) and metagenomic sequencing (**e**). (**f**) Gut microbial composition and function were analyzed to explore the association of gut microbiome with stereotypic behavior, and the gut-brain axis mechanism underlying stereotypic behavior in giant pandas.

In this study, we selected nine typical stereotypically behaving (SB) giant pandas and six normal pandas through behavioral observation. Their feces were collected for 16S ribosomal RNA (rRNA) gene sequencing (S1 Table in S1 File) of 145 fecal samples and metagenomic sequencing (S2 Table in S1 File) of 45 fecal samples to explore the association of gut microbiota with the SB giant pandas (Fig 1). Furthermore, we plan to identify the biomarker of SB giant pandas to provide a research basis for the mechanism of stereotypic behavior to promote the conservation of giant pandas. Our results reveal the association of gut microbiota with the stereotypic behavior in giant pandas, establishing a foundation to mitigate such behavior and enhance giant panda conservation efforts.

## Materials and methods

### Ethics statement

The animal research protocol was approved by Chengdu Research Base of Giant Panda Breeding Institutional Animal Care and Use Committee (protocol code 2018023, date of approval: October 2018).

### Animals and sample collection

To examine the gut microbiome of the stereotypically behaving (SB) giant panda, fifteen individuals were selected from over 200 captive giant pandas at the Chengdu Giant Panda Breeding Research Base through behavioral observations during long-term captive husbandry. All selected individuals had comparable dietary structures (bamboo as the main food, supplemented with a bit of bamboo shoots, panda cake, and apple), feeding environments (each giant panda was housed in an independent enclosure with comparable indoor and outdoor space), and health statuses (all individuals confirmed to be healthy by routine veterinary examinations). From September 2023 to February 2024, behavioral data were recorded over 10-day periods during 8:30–11:00 AM and 2:30–3:30 PM, totaling 604 hours (with an average of 40 hours per giant panda). Stereotypy criteria required the rigid performance of the same behavioral form three or more times consecutively (e.g., S1 File in S1 File) [6]. To ensure the reliability of observation, intra-observer and inter-observer reliability tests were conducted by two observers prior to formal observation. Intra-observer and inter-observer reliability were both assessed via Pearson correlation analysis on behavioral data obtained from seven 1-hour videos. For intra-observer reliability, data extracted by each observer twice at a one-week interval were compared; for inter-observer reliability, data extracted independently by the two observers were compared (see S2 File in S1 File for a detailed description of measuring reliability). The reliability analysis passed, as the correlation coefficients (r) for both intra-observer (r = 0.98, S3 Table in S1 File; r = 0.85, S4 Table in S1 File) and inter-observer reliability (r = 0.95; S5 Table in S1 File) exceeded 70% [22]. Based on observations during long-term captive husbandry, as well as pre-observation and formal observation using a continuous recording method by the two observers who passed the reliability tests, nine giant pandas were classified into the SB group and six into the normal group as controls (S1a Fig in S1 File), according to the stereotypy criteria. To account for age and gender effects on gut microbiota [23,24], giant pandas were further divided into six subgroups (S6 Table in S1 File).

A total of 145 feces were collected, with an average of about 10 feces per giant panda. After defecation, fresh feces were immediately collected, then quickly frozen in liquid nitrogen and maintained at −80 °C until analysis. The animal research was approved by Chengdu Research Base of Giant Panda Breeding Institutional Animal Care and Use Committee (Approval No. 2018023).

### Microbial genomic DNA extraction and 16S rRNA gene sequencing

Total microbial genomic DNA was extracted from fecal samples using the Genomic DNA extraction kit (Tiangen Biotech), adhering to the guidelines provided by the manufacturer. The primers 341F-CCTACGGGNGGCWGCAG and 806R-GGACTACNNGGGTATCTAAT were used to amplify the V3-V4 hypervariable region of the microbial 16S rRNA genes. Sequencing of the 16S rRNA genes was performed using the illumina NovaSeq 6000 platform to get 250-bp paired-end reads at Novogene Bioinformatics Technology Co., Ltd. All raw sequencing data were deposited in the National Genomics Data Center (NGDC), Genome Sequence Archive (GSA) database under the accession of CRA025077.

### 16S rRNA gene sequencing data analysis

After removing barcodes and primer sequences, FLASH (V1.2.11) was employed to merge paired-end reads [25]. Subsequently, quality filtering was performed using the fastp (Version 0.23.1) software [26], followed by chimera sequence detection against the Silva database (https://www.arb-silva.de/). The chimera sequences were removed using the vsearch package to get effective tags [27]. The effective tags were subsequently denoised using DADA2 within the QIIME2 software (Version QIIME2–202202), each de‐duplicated sequence generated after noise reduction using DADA2 is called Amplicon Sequence Variant (ASV) [28]. Based on the species annotations of ASVs by using a pretrained Naive Bayes classifier via QIIME2’s classify-sklearn algorithm, microbial abundance data for all taxonomic levels (kingdom to species) were yielded. We next filtered out all ASVs assigned to chloroplasts and mitochondria. Additionally, to correct biases caused by sequencing depth, the absolute abundance of ASVs was normalized using a standard of sequence number corresponding to the sample with the least sequences. Subsequently, all analyses were conducted using the normalized data.

To evaluate the complexity of species diversity, α-Diversity was calculated from Good’s-coverage, Shannon and Chao1 indices based on normalized ASVs in QIIME2. Differences in α-diversity indices between two groups were analyzed with the Wilcoxon rank sum test and among the six subgroups were analyzed with the Kruskal-Wallis test followed by the Conover-Iman post hoc test with Benjamini-Hochberg (BH) correction using R software (Version 4.0.3). We used β-diversity based on unweighted Unifrac distance to evaluate differences in samples, calculated by QIIME2 software. Principal Coordinate Analysis (PCoA) was conducted to visualize intricate, multidimensional data using ade4 and ggplot2 packages within R software. Permutational multivariate analysis of variance (Adonis) analysis based on unweighted Unifrac distance was used to analyze microbial community structural differences among the groups via the vegan package in R software.

Biomarkers were identified through Linear Discriminant Analysis (LDA) Effect Size (LEfSe) using LEfSe software (Version 1.0) [29], which uses the parameter “LDA score > 2” to detect species that exhibit significant differences in abundance between different groups.

### Metagenomic sequencing and data analysis

Forty-five fecal samples (three samples per giant panda) were selected (S2 Table in S1 File) for metagenomic sequencing. After the library passed the quality check, various libraries were pooled based on their target data output criteria and valid concentrations, and then subjected to 150-bp paired-end sequencing using NovaSeq X Plus platform at Novogene Bioinformatics Technology Co., Ltd. All raw sequencing data were deposited in the GSA database under the accession of CRA025077.

Sequences were first quality controlled with Fastp (Version 0.23.1). A total of 566.75 gigabytes (Gb) of sequence data (with an average of ~12.59 Gb per sample) (S2 Table in S1 File) were obtained. To filter out possible DNA contamination from the host, we aligned the sequence data with the genomes of the giant panda and Moso bamboo (*Phyllostachys edulis*), and 510.72 Gb (with an average depth of ~11.35 Gb per sample) of clean data remained by using Bowtie2 software (Version 2.2.4) for subsequent analyses [30–32]. Clean data were assembled using MEGAHIT software (Version 1.2.9) [31,33], with subsequent processing to generate scaftigs without N by fragmenting the resulted scaffolds from the N junction [34,35]. With the default parameters, MetaGeneMark (Version 2.1) was employed to predict Open Reading Frames for the scaftigs (≥ 500 bp) of each sample [30,36–39]. The initial gene catalogue was generated by removing redundancy in the Open Reading Frames [40] through the use of CD-HIT software (Version 4.5.8) [41,42], employing the following parameter settings: -c 0.95, -G 0, -aS 0.9, -g 1, -d 0 [37,39]. Bowtie2 was used to quantify the initial gene catalogue [34,37], and genes that had two or fewer reads in each sample were excluded to ultimately generate the unigenes for further analysis [40]. Subsequently, DIAMOND software (Version 2.1.6) [43] was employed to align unigene sequences against the microNR database (https://www.ncbi.nlm.nih.gov/), utilizing a parameter setting of 1e-5 [31]. From the results of the alignments for each sequence, the one with evalue ≤ min. evalue*10 was chosen. Given that multiple alignment results might exist for each sequence, the Lowest Common Ancestors algorithm, incorporated in MEGAN software, was utilized to ascertain the species annotation information of the sequence [44]. LEfSe analysis (LDA Score is 2 by default) was used to discover biomarkers at the species level that differ significantly among subgroups using LEfSe software [29]. When appropriate, we used the Wilcoxon rank sum test in R software.

To quantify the functional diversity of the microbial community, metagenomic sequences were functionally annotated through alignment of unigenes against the Kyoto Encyclopedia of Genes and Genomes (KEGG) database (http://www.kegg.jp/kegg/) by using DIAMOND software [45,46]. Only the best blast hit results derived from sequence alignments were selected for subsequent analysis [37,39,47,48]. LEfSe analysis was completed using LEfSe software to find microbial function differences among subgroups. When appropriate, we used Wilcoxon rank sum test in R software.

## Results

### Consistent dominant gut microbiota composition in SB and normal giant pandas

Based on the behavioral observation, nine giant pandas were classified into the stereotypically behaving (SB) group, and six into the normal group as controls (S1a Fig in S1 File). As age and gender have a significant influence on the gut microbiota of giant pandas [23,24], they were further divided into six subgroups: SB geriatric females (SGF); normal geriatric females (NGF); SB adult males (SAM); normal adult males (NAM); SB adult females (SAF); and SB geriatric males (SGM) (S6 Table in S1 File). NGF and NAM served as controls for SGF and SAM, respectively.

**Fig 2:**
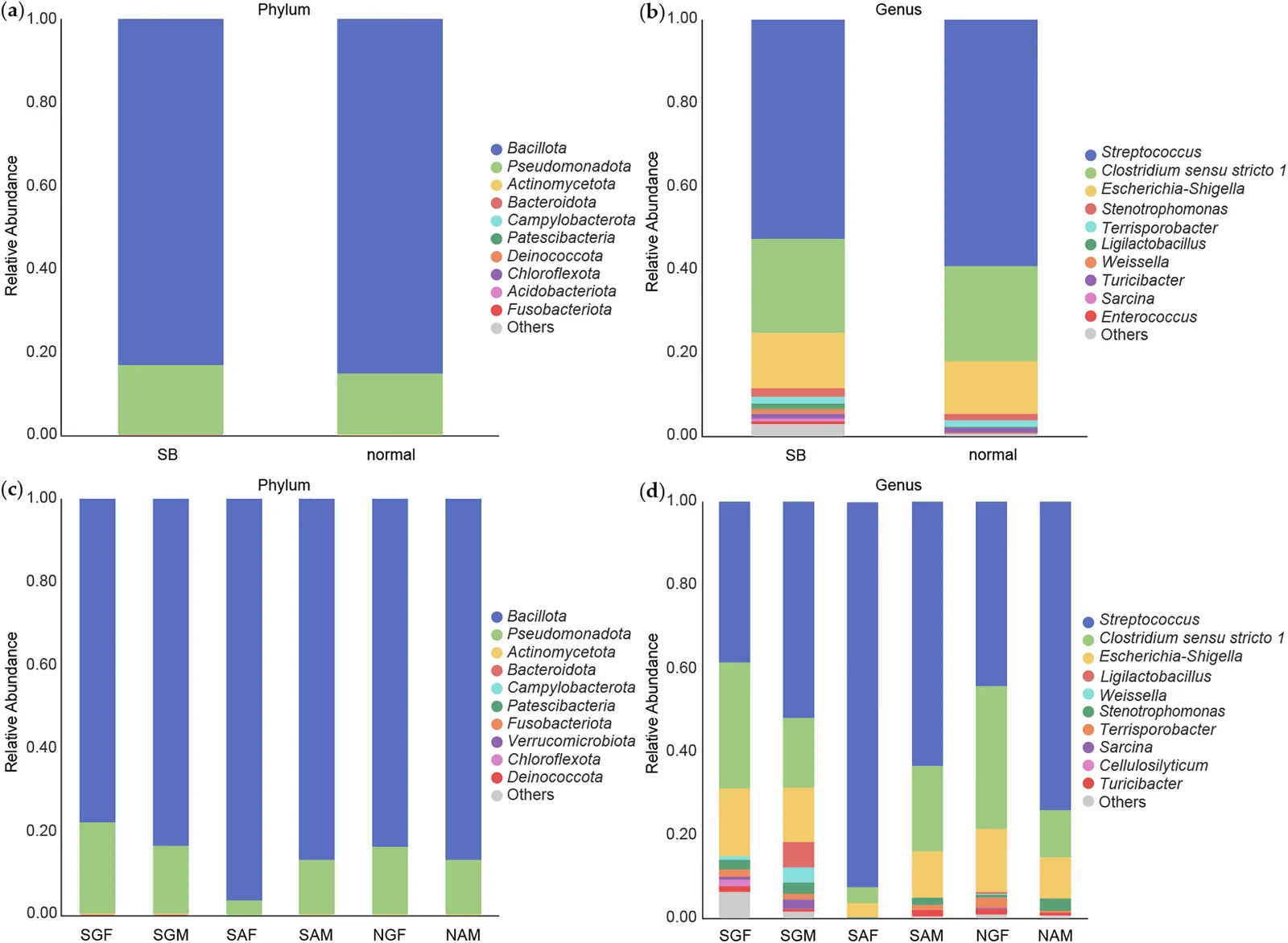
Gut microbiome composition of giant panda. Panels (**a**) and (**b**) present data from the SB group-normal group, while panels (**c**) and (**d**) show the six subgroups: SB geriatric females (SGF); SB geriatric males (SGM); SB adult females (SAF); SB adult males (SAM); normal geriatric females (NGF); and normal adult males (NAM). Bar charts display the relative abundance of 16S rRNA gene sequencing at the phylum and genus levels.

From 145 fecal samples, the analysis of 16S rRNA gene sequencing yielded a total of 14,352,031 high‐quality sequences, averaging 98,979 reads per sample (S1 Table in S1 File), and the samples demonstrated an average Good’s coverage of 99.99%, with relative stability (S1b Fig in S1 File). Additionally, the curves of Rank Abundance (S1c Fig in S1 File) displayed a gradual decline, which suggested that the sample size was adequate, and the sequencing depth adequately reflected the comprehensive microbiome profile of fecal samples from giant pandas. A total of 3,463 ASVs were produced with a 100% similarity clustering. After removing ASVs assigned to chloroplasts and mitochondria, the shared ASV numbers of the six subgroups were 47, accounting for 1.48% of the total 3,179 ASVs (S1d Fig in S1 File), showing that there were large differences among them. We classified the ASVs into 208 species, 384 genera, 185 families, 111 orders, 49 classes, and 29 phyla. *Bacillota* was the most dominant phylum in the gut microbiota of both SB and normal giant pandas, followed by *Pseudomonadota* and *Actinomycetota* (Fig 2a, 2c). At the genus level, the gut microbiota of SB and normal giant pandas was both primarily composed of *Streptococcus*, followed by *Clostridium sensu stricto 1* and *Escherichia-Shigella* (Fig 2b, 2d).

### Gut microbial diversity differs significantly between SB and normal giant pandas

**Fig 3:**
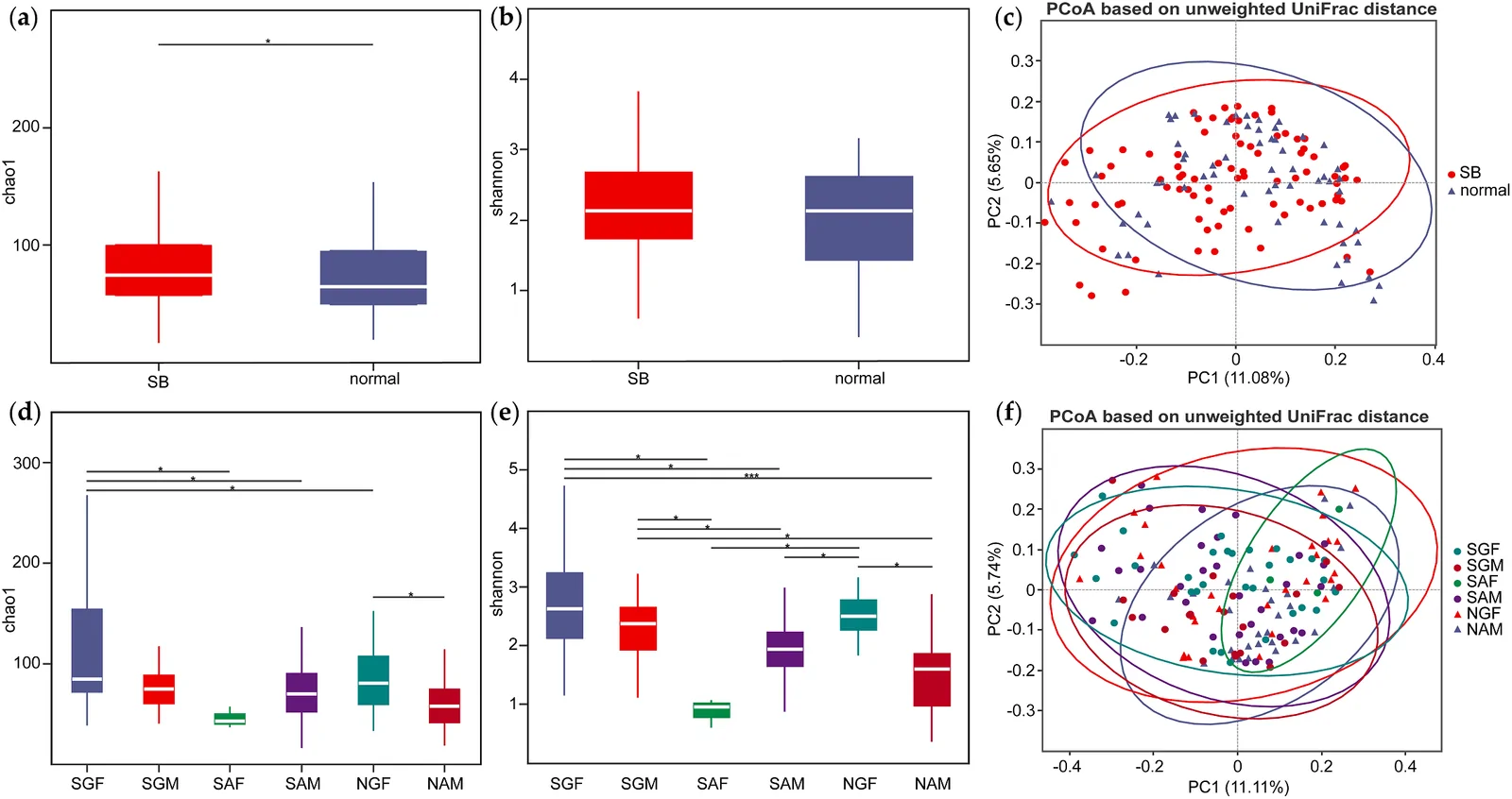
Gut microbial diversity of captive giant pandas. Boxplots show Chao1 (**a**) and Shannon (**b**) indices comparing SB and normal groups (Wilcoxon rank-sum test), as well as Chao1 (**d**) and Shannon (**e**) indices across six subgroups (Kruskal–Wallis test followed by Conover–Iman post hoc with Benjamini–Hochberg (BH) correction). *p < 0.05, **p < 0.01; ***p < 0.001. Principal coordinate analysis (PCoA) plots for SB and normal groups (**c**) and six subgroups (**f**) based on unweighted Unifrac distance analysis.

Analysis of the Chao1 and Shannon indices showed that the SB group exhibited higher α-diversity than the normal group, and the difference between Chao1 indices was significant (*p* = 0.038, Wilcoxon rank-sum test; Figs 3a and 3b). Through the PCOA based on unweighted UniFrac distance, a separation between the SB and normal groups was found (Fig 3c), and Adonis also demonstrated that there was a significant difference in gut microbiota composition between the SB and normal groups (Adonis, *p* = 0.002; S7 Table in S1 File).

Then the six subgroups were further analyzed as age and gender have a significant effect on the gut microbiota of giant pandas [23,24]. Analysis of the Chao1 and Shannon indices showed that there were significant differences among the six subgroups (*p* < 0.001, Kruskal-Wallis test). Many (13 of 30) of the pairwise comparisons between subgroups showed significant differences (*p* < 0.05, post hoc in Conover–Iman test with BH correction). The SB subgroups exhibited higher α-diversity than the gender/age-matched normal subgroups, but the differences were not statistically significant (*p* > 0.05) (S8 Table in S1 File; Figs 3d and 3e). Through the PCoA based on unweighted Unifrac distance (Fig 3f), we found SGF vs. NGF (*p* = 0.045, Adonis) and SAM vs. NAM (*p* = 0.004, Adonis) were significantly different in gut microbiota (S7 Table in S1 File).

### Identification of gut microorganisms associated with stereotypic behavior in giant pandas

**Fig 4:**
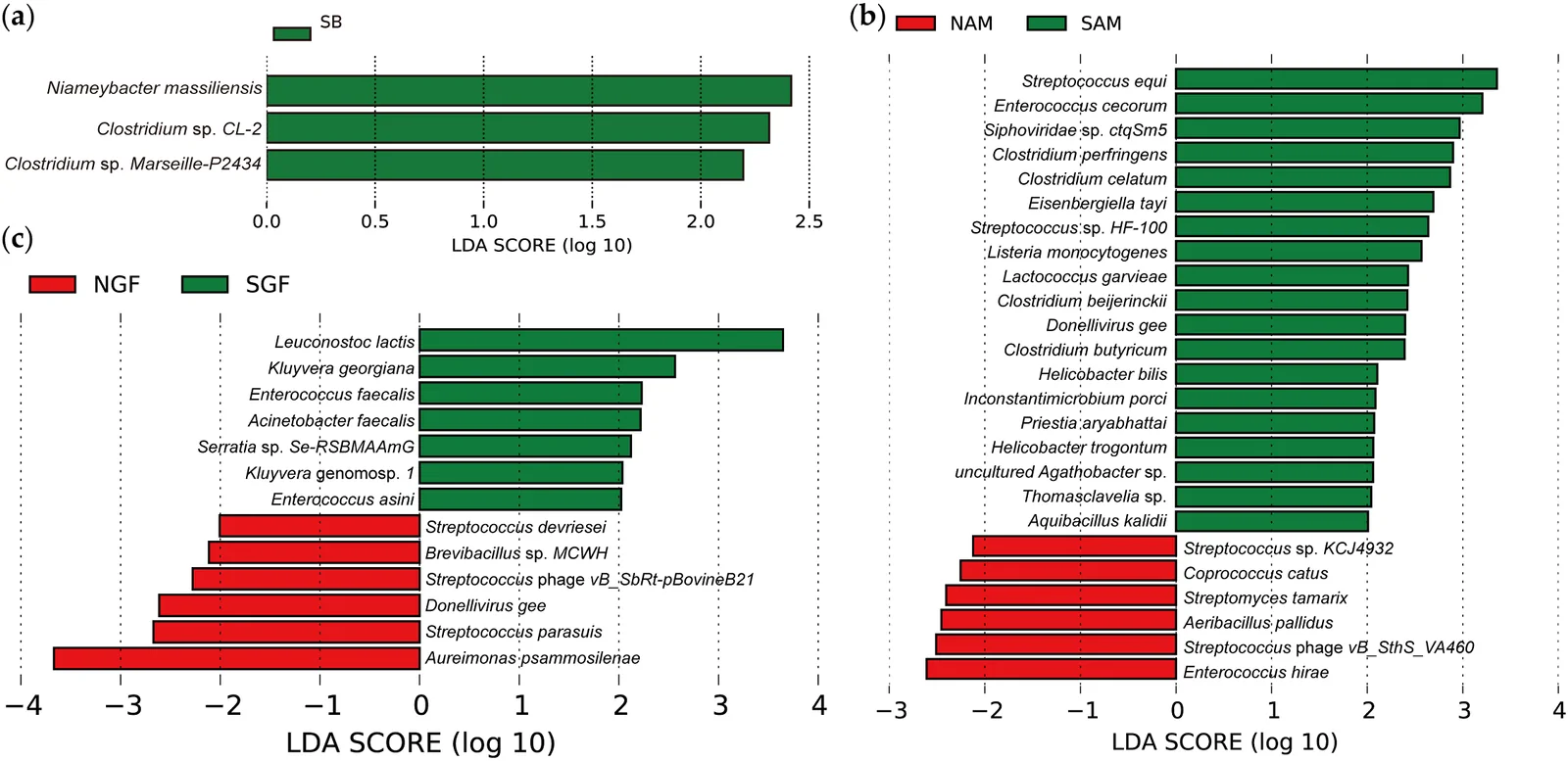
Linear discriminant analysis (LDA) effect size (LEfSe) analysis identifying taxa with differential abundance between groups (LDA > 2, *p* < 0.05). SB vs. normal groups based on 16S rRNA gene sequencing data (**a**); NAM vs. SAM (**b**) and NGF vs. SGF (**c**) subgroups based on metagenomic data.

To compare the gut microorganismal species between SB and normal giant pandas, LEfSe analysis (*p* < 0.05, LDA > 2) was applied to the 16S rRNA gene profiles (Fig 4a). *Clostridium* sp. *CL-2*, *Clostridium* sp. *Marseille-P2434*, and *Niameybacter massiliensis* were significantly enriched in the SB group.

Although LEfSe analysis of the metagenomic profiles (S2a Fig in S1 File) revealed no common biomarker shared by SAM and SGF compared with NAM and NGF subgroups (Fig 4b, 4c), shared abundance trends were found. At the species level, *Serratia* sp. *Se-RSBMAAmG* was identified as the biomarker between SGF and NGF, which was significantly enriched in SGF, and the abundance of *Serratia* sp. *Se-RSBMAAmG* was also significantly higher in SAM than in NAM (*p* = 0.031, Wilcoxon rank-sum test; S3a Fig in S1 File). The abundance of *Aureimonas psammosilenae* was significantly lower in SGF, with a similar but non-significant trend in SAM (*p* = 0.402, Wilcoxon rank-sum test; S3b Fig in S1 File). *Clostridium celatum*, *Clostridium beijerinckii*, *Clostridium butyricum*, *Lactococcus garvieae* were significantly enriched in SAM, with similar but non-significant trends in SGF (*p* > 0.05, Wilcoxon rank-sum test; S3c-S3f Figs in S1 File); The abundance of *Enterococcus hirae* was significantly lower in SAM, with a similar but non-significant trend in SGF (*p* = 0.453, Wilcoxon rank-sum test; S3g Fig in S1 File). Notably, *Clostridium* species were identified in 16S rRNA gene and metagenomic sequencing results.

### Analysis of gut microbial functions associated with stereotypic behavior in giant pandas

To explore the association between gut microbiome and the mechanism underlying stereotypic behavior of giant pandas, we employed the KEGG database in the metagenome profile.

**Fig 5:**
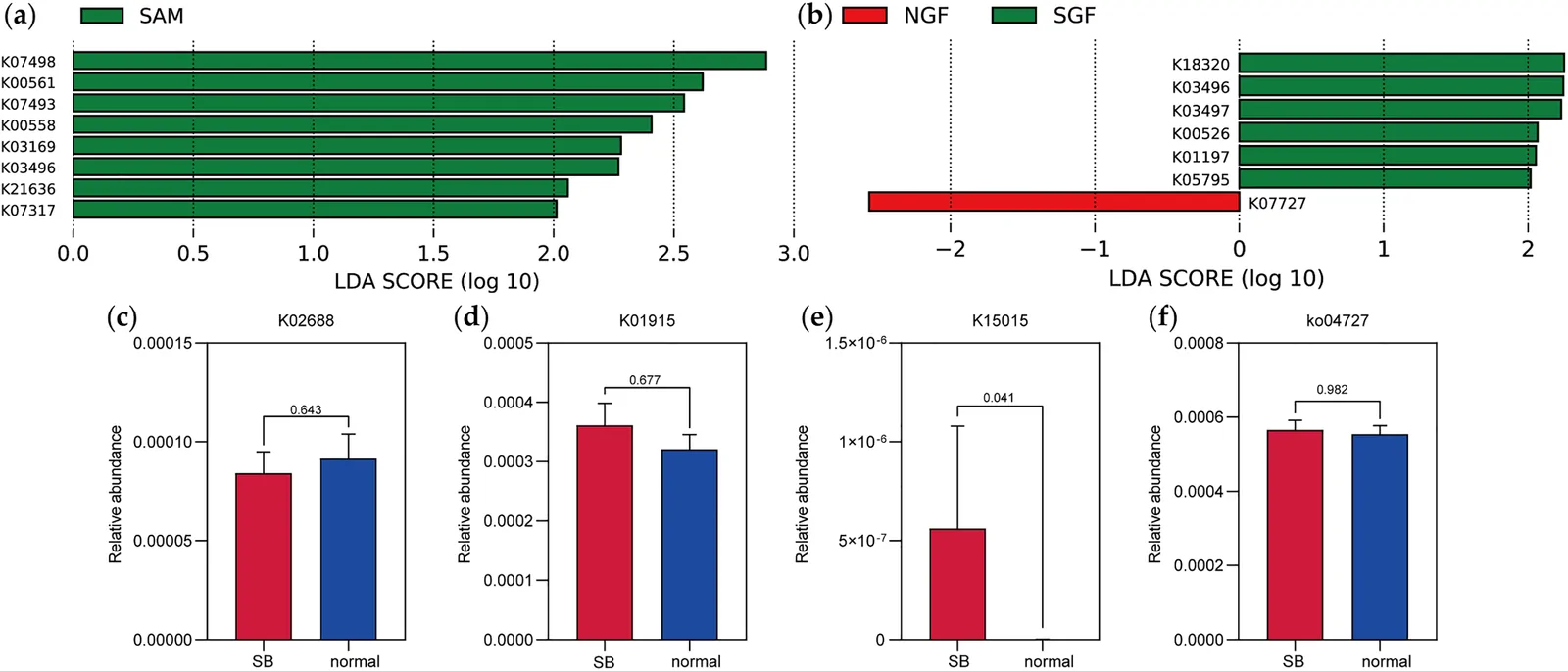
Analysis of Kyoto Encyclopedia of Genes and Genomes (KEGG) ortholog groups (KOs) and the KEGG pathway of the gut microbiomes between groups. LEfSe analyzed the histogram of LDA (*p* < 0.05, LDA > 2) value distributions of SAM and NAM (**a**) and SGF and NGF (**b**) subgroups. Comparison of the relative abundance of KOs between SB and normal groups. The KOs related to propionate catabolism (**c**), glutamine synthesis (**d**), and GABA exocytosis of presynaptic terminal (**e**). (**f**) The GABAergic synapse pathway (Wilcoxon rank sum test, data are presented as mean ± SEM).

LEfSe analysis (*p* < 0.05, LDA > 2; Fig 5a, 5b) was conducted between the SB subgroups and gender/age-matched normal subgroups to elucidate functional disparities according to the KEGG ortholog groups (KOs) encoded by the microbial genes. The KO (K03496) related to chromosome partitioning protein was the common functional biomarker in both SGF and SAM versus NGF and NAM. The KO (K07493) related to putative transposase, and the KO (K00558) related to DNA (cytosine-5)-methyltransferase 1, which plays a role in several diseases such as cancer and neurological diseases [49] and mapped to *Clostridium* species (*C. beijerinckii*, *C. celatum* and *C. butyricum*) that tended to be enriched in the gut microbiota of SB giant pandas, were significantly enriched in SAM, with similar but non-significant trends in SGF (*p* > 0.05, Wilcoxon rank-sum test; S4a, S4b Fig in S1 File).

Furthermore, the KOs were compared between the SB and normal groups with the Wilcoxon rank-sum test. The abundance of KO (K02688), which is the sole KO involved in propionate catabolism encoded by the microbial genes in the KEGG database, showed a lower but non-significant trend in the SB group (*p* = 0.643; Fig 5c). The KO (K01026) related to propionate CoA-transferase, which is involved in propionate formation [50], was mapped to *C. beijerinckii* and *C. butyricum*.

KO (K01915), which was related to glutamine synthetase and mapped to *C. beijerinckii* and *C. celatum*, showed a more abundant but non-significant trend in the SB group (*p* = 0.677; Fig 5d). The abundance of KO (K15015), which is involved in gamma-aminobutyric acid (GABA) exocytosis of the presynaptic terminal, was significantly higher in the SB group (*p* = 0.041; Fig 5e). Both KO (K01915) and KO (K15015) are related to GABA secretion and participate in the GABAergic synapse pathway (ko04727), which also showed a more abundant but non-significant trend in the SB group (*p* = 0.982; Fig 5f).

## Discussion

This research explored the composition, diversity, and function of the gut microbiota in stereotypically behaving (SB) giant pandas to elucidate their potential association with stereotypic behavior. Preliminarily, we found that SB giant pandas and normal giant pandas had similar dominant microbiota composition. This finding was consistent with previous reports on ASD [51,52], which is characterized by stereotypic behavior as an iconic symptom [8,9]. However, our findings reveal significant alterations in the gut microbiota of SB giant pandas. Similar alterations have also been observed in other captive wild animals displaying stereotypic behavior [53,54]. Moreover, we confirmed the elevation of microbial richness in SB giant pandas compared with that in normal giant pandas, which was consistent with previous reports on ASD [20,55], implying an imbalance of gut microbiota in SB giant pandas, and the extra parts of gut microbiota may contain the species that associate with stereotypic behavior. Thus, it is necessary to focus on further study of potential microbiological factors.

We found eight species that may be closely associated with the stereotypic behavior of giant pandas, most of which were *Clostridium* bacteria. First, *Clostridium* sp. *CL-2*, *Clostridium* sp. *Marseille-P2434*, *C. celatum*, *C. beijerinckii*, and *C. butyricum* showed a trend of enrichment in SB giant pandas. It has been reported that the growth of *Clostridium* bacteria in ASD children has a harmful effect on the gut-brain axis [18]. Similarly, significantly higher *Clostridium* abundance has been observed in captive forest musk deer than in wild populations [56], suggesting that gut microbial alterations may be associated with environmental stress in a captive environment. Second, *L. garvieae* also showed a trend of enrichment in SB giant pandas. *Lactococcus garvieae*, a pathogen, can induce inflammatory cell infiltration [57], a process closely associated with ASD [58]. Notably, *Serratia* sp. *Se-RSBMAAmG*, a species that has not been reported previously, was significantly enriched in the gut microbiota of SB giant pandas across genders and ages, suggesting that it may be a potential biomarker of stereotypic behavior in giant pandas. Finally, the abundance of *E. hirae*, a probiotic with bactericidal effects against enteric pathogens via bacteriocin production [59–61], showed a lower trend in the gut of SB giant pandas than in controls. These results support the hypothesis that the stereotypic behavior of giant pandas may be associated with the elevation of *Serratia* sp. *Se-RSBMAAmG*, *Clostridium* bacteria and *L. garvieae*, as well as the reduction of probiotic *E. hirae*, in the intestine. However, the mechanism of these species underlying the stereotypic behavior requires further exploration.

**Fig 6:**
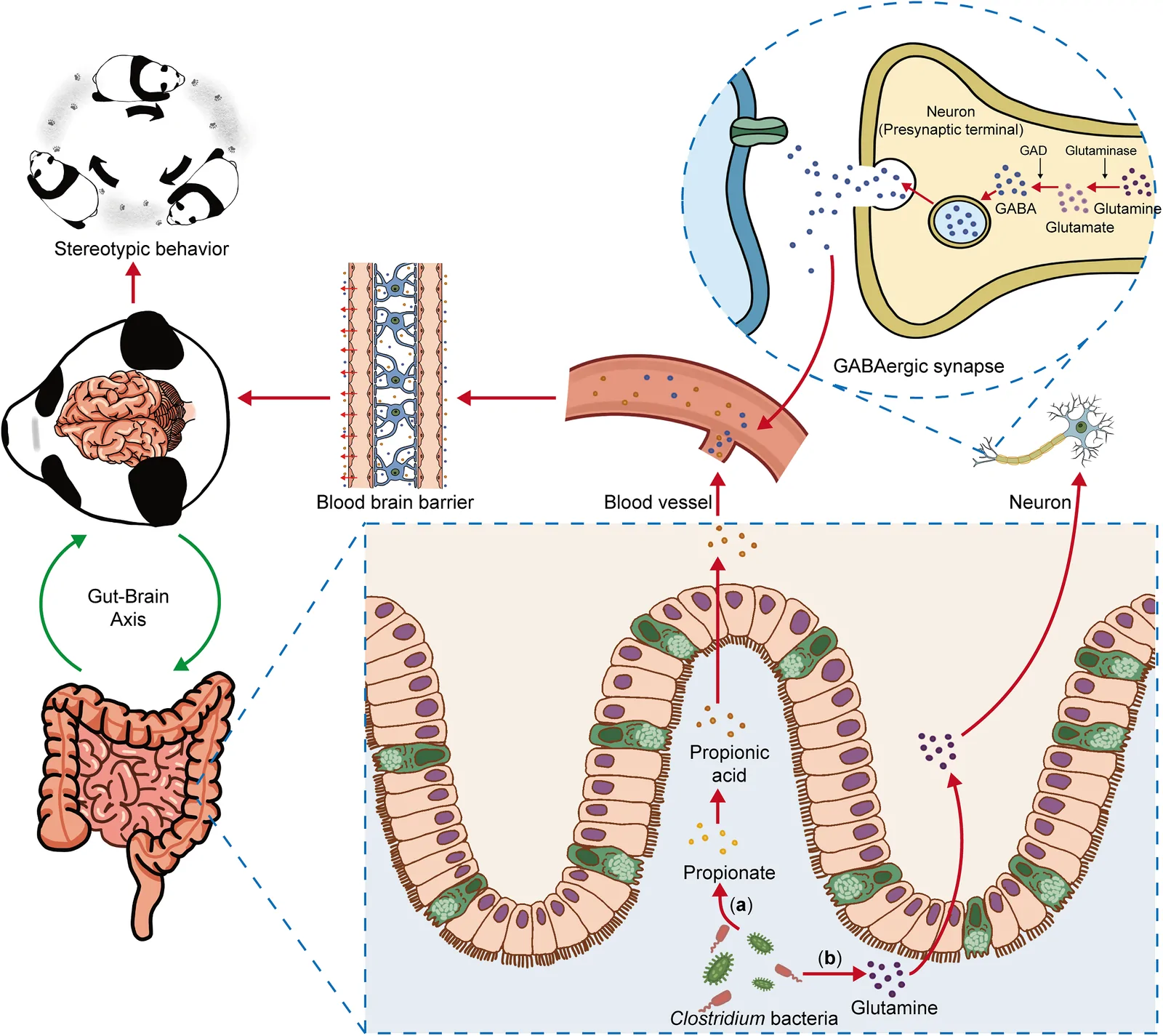
Schematic diagram of the *Clostridium* bacteria-mediated gut-brain axis hypothesis underlying stereotypic behavior in giant pandas. (**a**) Elevated *Clostridium* bacteria abundance enhances propionate synthesis while reducing its catabolism in the intestine, resulting in elevated propionic acid levels, then propionic acid enters blood circulation and reaches the brain, potentially inducing the emergence of stereotypic behavior. (**b**) Elevated *Clostridium* bacteria abundance enhances glutamine synthesis, providing precursors for neuronal GABA production via the GABAergic synapse pathway, then GABA enters blood circulation and reaches the brain, potentially inducing the emergence of stereotypic behavior.

The enrichment trend of the *Clostridium* bacteria in the gut microbiome, accompanied by the dysregulation of associated metabolic activities, may be the reason for stereotypic behavior of giant pandas. We found that the functional genes related to propionate catabolism showed a trend of lower abundance in SB giant pandas than in normal giant pandas, implying elevated levels of propionate in SB giant pandas. Propionate is a derivative of propionic acid, linked to neurodevelopmental disorders, including ASD [62–64]. Intraventricular injection of propionic acid in rats can lead to significantly greater stereotypic behavior than in control rats [65]. Traditionally, elevated propionate levels are associated with enhanced biosynthesis by *Clostridium* genus [51]. It has been reported that maternal deprivation during the early life of captive giant pandas, a key determinant of abnormal behavior in stress model individuals, increased the relative abundance of *Clostridium* bacteria, which is significantly and positively correlated with propionic acid metabolism [66]. Interestingly, we observed that five *Clostridium* species were more abundant in the gut microbiota of SB giant pandas than in normal giant pandas, and two of them were mapped by the functional genes related to propionate formation [50]. Moreover, two *Clostridium* species mentioned above were mapped by the functional genes related to glutamine synthesis that provides precursors for neuronal GABA production via the GABAergic synapse pathway. These functional genes showed an upregulation trend in SB giant pandas. Additionally, the abundance of functional genes associated with GABA exocytosis of the presynaptic terminal was significantly higher in SB giant pandas than in normal giant pandas, further implying the elevated levels of GABA. GABA, an important neurotransmitter that regulates central nervous system activity, can mediate the interaction between gut microbiota and mental health, and imbalanced GABA metabolism is a notable characteristic of gut microbiota in ASD patients [67,68]. Our results further support the hypothesis that the enhanced abundance of *Clostridium* bacteria in the gut microbiome, accompanied by elevated levels of propionate and GABA, may be associated with the emergence of stereotypic behavior in giant pandas through the gut-brain axis (Fig 6). More research is needed to verify this hypothesis. The primary limitation of this study is the relatively small number of subjects, which inevitably limits the statistical power and heightens the risk of false-positive associations. However, this limitation stems from our rigorous selection of subjects, which ensured the reliability of our results. To mitigate concerns about this limitation, we performed repeated sampling on each individual (over 10-day behavioral observations and approximately 10 fecal samples per giant panda), which improved statistical power. We also performed stratified analyses to control for confounding factors, and we applied stringent BH corrections for multiple comparisons. In addition to this primary limitation, the observed associations between specific microbes and stereotypic behavior are merely correlational, not causal. The underlying mechanisms by which these microbes may contribute to the development of stereotypic behavior remain speculative. Given these limitations, our research should be viewed as an exploratory pilot study. Therefore, studies with larger sample sizes and further experiments are needed to validate these preliminary findings. Nevertheless, this research identifies candidate bacteria and proposes potential mechanistic pathways, laying a foundation for future targeted interventions and therapeutic strategies for stereotypic behavior.

## Conclusion

Our research offers a comprehensive profile of the gut microbiome of stereotypically behaving (SB) giant pandas, and differences in gut microbial diversity, composition, and function between SB and normal giant pandas were identified. Our results indicate that there is gut microbial dysbiosis in SB giant pandas, and the stereotypic behavior of giant pandas may be related to the elevation of *Clostridium* bacteria such as *Clostridium* sp. *CL-2*, *Clostridium* sp. *Marseille-P2434*, *C. celatum*, *C. beijerinckii*, and *C. butyricum*, as well as *Serratia* sp. *Se-RSBMAAmG* and *L. garvieae*, and the reduction of probiotic *E. hirae* in the intestine. Moreover, the *Clostridium* bacteria may affect propionate metabolism and the GABAergic synapse pathway, potentially affecting the emergence of stereotypic behavior. Our findings contribute to the evidence that links the gut microbiome to stereotypic behavior of giant pandas. Identifying the specific bacteria associated with stereotypic behavior of giant pandas and elucidating their mechanistic pathways provides foundations for targeted interventions and therapeutic approaches.

## Supporting information

S1 Fig Analysis of the duration of stereotypic behavior and the depth and diversity of gut microbiome sequencing in captive giant pandas. (**a**) The duration of stereotypic behavior in stereotypically behaving (SB) vs. normal groups (data are presented as mean ± SEM, ***p < 0.001, Wilcoxon rank sum test). Good’s coverage index rarefaction curve (**b**) reflects the sequencing adequacy of samples, and rank abundance curve (**c**) shows the richness and evenness of species. (**d**) Venn diagram illustrating the overlap of ASVs across six subgroups.
(TIF)

S2 Fig The rarefaction curves of the core genes (a) and pan genes (b) indicated that the sequencing results could encompass the real genes in the tested samples, fulfilling the prerequisites for further analyses. A total of 1,068,409 unigenes were generated, and the total length was 580.71Mbp, with an average length of 543.53 bp.
(TIF)

S3 Fig Differences in the relative abundance of various gut microbes between the SB and the gender/age-matched normal subgroups. The relative abundance of *Serratia* sp. *Se-RSBMAAmG* (a), *Aureimonas psammosilenae* (b) in SB adult males (SAM) and and normal adult males (NAM) subgroups. The relative abundance of *Clostridium celatum* (c), *Clostridium beijerinckii* (d), *Clostridium butyricum* (e), *Lactococcus garvieae* (f) and *Enterococcus hirae* (g) in SB geriatric females (SGF) and normal geriatric females (NGF) subgroups (Wilcoxon rank sum test, data are presented as mean ± SEM).
(TIF)

S4 Fig Differences in the relative abundance of two KOs of the gut microbiomes between SGF and NGF subgroups. (**a**) The KO related to putative transposase. (**b**) The KO related to DNA (cytosine-5)-methyltransferase 1 (DNMT1) (Wilcoxon rank sum test, data are presented as mean ± SEM).
(TIF)

S1 Table 16S ribosomal RNA (rRNA) Sequencing data summary of 145 samples. (XLS)

S2 Table Metagenomic Sequencing data summary of 45 samples. (XLS)

S3 Table Intra-observer reliability of observer A. (XLS)

S4 Table Intra-observer reliability of observer B. (XLS)

S5 Table Inter-observer reliability between observer A and observer B. (XLS)

S6 Table The details and grouping of giant pandas in the study. (XLS)

S7 Table The results of Permutational multivariate analysis of variance (Adonis) based on unweighted Unifrac distance between groups. (XLS)

S8 Table *P* values of post hoc in Conover-Iman test with Benjamini-Hochberg (BH) correction of each comparison for two alpha diversity indices. (XLS)

S1 File A video of typical stereotypic behavior of the giant panda recorded during the sampling process. (MP4)

S2 File Definition of stereotypic behavior; Measuring reliability of observation. (DOC)
