# Correction: Gut microbiome is associated with stereotypic behavior of the giant panda (*Ailuropoda melanoleuca*)

**DOI:** 10.1371/journal.pone.0361345

**Published:** 2026-10-21

**Authors:** Anqi Hu, Jinyuan Liu, Peng Chen, Kuixing Yang, Fan Xu, Gai Luo, Jiali Zhang, Mengnan He

The Funding statement for this article is incorrect. The correct Funding statement is as follows: PC was supported by the Chengdu Research Base of Giant Panda Breeding (Grant No. 202503KY0004) - https://www.panda.org.cn/. FX was supported by the National Natural Science Foundation of China (Grant No. 32270552) - https://www.nsfc.gov.cn/, the National Key Research and Development Program of China (Grant No. 2023YFE0108400) - https://www.most.gov.cn/index.html, and the Key Discipline Project at the School of Public Health, Chengdu Medical College (Grant No. 21) - https://www.cmc.edu.cn/. The funders had no role in study design, data collection and analysis, decision to publish, or preparation of the manuscript.
