# Supplementary material for: Gut microbiome is associated with stereotypic behavior of the giant panda (Ailuropoda melanoleuca)
Source: PLoS One. 2026 Sep 23;21(9):e0357625. doi: 10.1371/journal.pone.0357625 (PMC13600500; doi:10.1371/journal.pone.0357625)
Supplement: Supplementary file 1 [file pone.0357625.s014.doc]

Definition of stereotypic behavior

Stereotypy criteria required the rigid performance of the same behavioral form three or more times consecutively (e.g., S1 File) [6] and/or was embedded in the stereotypic ethogram established in our previous study (S9 Table) [69] in which it was repeated at least three times.

**S9 Table. Stereotypic ethogram of captive giant panda [69]**

| Behaviour | Description |
| --- | --- |
| Stereotypic pacing | Repeatedly walking or circling on a fixed path,moving in a stereotypic pattern,which is recorded as stereotypic pacing only when walking at least three times on the same path. |
| Head toss | Suddenly raising the head,and then whipping it upward or sideways in a whipping motion.This behaviour is often accompanied by stereotypic pacing. |
| Ruck | Constantly changing the center of gravity of the body between the left and right sides,while maintaining a fixed posture.Or standing up and swinging head,shoulders or even whole body from side to side in a fixed position. |
| Stereotypic somersault | Full or truncated somersault.Full somersault involves placing head on ground and pushing hindquarters over the head in a complete revolution.Truncated somersault involves placing head to ground as if to do a somersault and touching shoulders to the ground but does not complete action. |
| Lahaleeb vertically | Standing on hind legs,the upper body is vertical upward,can use the surrounding support to maintain balance. |
| Stretch tongue | Protruding the tongue from the mouth and flexing constantly. |
| Stereotypic scratching | Animal scratches itself repeatedly;form is rigid and scratch directed to same location on body (not grooming). |
| Paw suck | Repetitive,sustained sucking of the toes/paw,sucking sound is often heard. |
| Climb cage | The front paws lying on the door or window railings,the hind legs standing on the ground,and the forelimbs showing an upward climbing posture. |
| Lick claw | Constantly licking all kinds of objects with the tongue, such as grass,wood and claws. |
| Teeth clatter | Repeatedly opening the upper and lower teeth,and the knocking sound can be heard. |

Measuring reliability of observation

This study was observed by two observers. Before the formal observation, the two observers memorized the stereotypic ethogram (Table S9) and repeatedly watched the video of giant panda stereotypic behavior to train them to be familiar with giant panda stereotypic behavior. In order to prove the reliability of the observation, the reliability analysis was performed on the two observers. The reliability analysis can be distinguished: intra-observer reliability test and inter-observer reliability test. The intra-observer reliability was determined by Pearson correlation analysis of the behavioral data obtained by each observer who repeatedly observed and recorded seven 1-hour videos at intervals of one week on different occasions. The inter-observer reliability was evaluated by comparing the behavioral data recorded by the two observers from the same seven 1-hour videos, also using Pearson correlation analysis. The seven 1-hour videos were recorded from different stereotypically behaving giant pandas respectively. All behavioral data were recorded as duration. The duration of behavior was the percentage of time spent on performing stereotypic behavior within one hour. Formal observations commenced only after the correlation coefficients (r) for both intra-observer (S3 and S4 Tables) and inter-observer reliability (S5 Table), calculated using R software, exceeded 70% [22].

**S3** Table. Intra-observer reliability of observer A

| Session | 1 | 2 | 3 | 4 | 5 | 6 | 7 |
| --- | --- | --- | --- | --- | --- | --- | --- |
| Duration(s·h^-1^) |  |  |  |  |  |  |  |
| Occasion A | 1780 | 1014 | 1695 | 568 | 1259 | 604 | 482 |
| Occasion B | 2008 | 910 | 1668 | 759 | 1360 | 651 | 477 |

The intra-observer reliability of observer A expressed as a Pearson correlation coefficient (r) is + 0.98 (n = 7; 5 d.f.).

**S4** Table. Intra-observer reliability of observer B

| Session | 1 | 2 | 3 | 4 | 5 | 6 | 7 |
| --- | --- | --- | --- | --- | --- | --- | --- |
| Duration(s·h^-1^) |  |  |  |  |  |  |  |
| Occasion A | 1853 | 1062 | 1739 | 726 | 922 | 547 | 651 |
| Occasion B | 1141 | 1062 | 1739 | 726 | 690 | 559 | 242 |

The intra-observer reliability of observer B expressed as a Pearson correlation coefficient (r) is + 0.85 (n = 7; 5 d.f.).

**S5** Table. Inter-observer reliability

| Session | 1 | 2 | 3 | 4 | 5 | 6 | 7 |
| --- | --- | --- | --- | --- | --- | --- | --- |
| Duration(s·h^-1^) |  |  |  |  |  |  |  |
| Observer A | 1780 | 1014 | 1695 | 568 | 1259 | 604 | 482 |
| Observer B | 1853 | 1062 | 1739 | 726 | 922 | 547 | 651 |

The inter-observer reliability expressed as a Pearson correlation coefficient (r) is + 0.95 (n = 7; 5 d.f.).

SI Reference

[69] Hu A, Chen P, Zha J, Zhang H, Luo G, Zhang J, et al. Study on stereotypic behaviour of captive giant pandas (Ailuropoda melanoleuca). Prog Vet Med. 2025;46(12):94–8. https://doi.org/10.16437/j.cnki.1007-5038.2025.12.005
