# Supplementary material for: Gut microbiome is associated with stereotypic behavior of the giant panda (Ailuropoda melanoleuca)
Source: PLoS One. 2026 Sep 23;21(9):e0357625. doi: 10.1371/journal.pone.0357625 (PMC13600500; doi:10.1371/journal.pone.0357625)
Supplement: Supplementary file 2 [file pone.0357625.s015.doc]

Response to Editor and Reviewers

Dear Editor and Reviewers,

We genuinely appreciate you for taking the time to review our manuscript and provide valuable comments. Your insightful comments have significantly contributed to the improvements in the current version. We have carefully considered each comment and have made every effort to address them. The following is a summary of the major revisions in the revised manuscript:

1.	**Response to concerns regarding the sample size:** We have acknowledged the small sample size as a limitation, clarified that this limitation stems from our rigorous selection of subjects, and supported the robustness of our conclusions by highlighting our sampling strategy, application of Benjamini–Hochberg correction, and consistency with previous studies. We have also explicitly defined this study as an exploratory pilot study in the revised Discussion section.

2.	**Improved methodological transparency:** We have expanded the description of controlled variables and incorporated condensed reliability testing procedures from the Supporting Information into the Methods section.

3.	**Expanded Discussion with cross-species comparisons:** We have added new references and discussions comparing our findings with microbiome studies in other captive wildlife species.

4.	**Improved readability:** We have simplified the figure legends, corrected grammatical and typographical errors, clearly defined the abbreviation "SB", and italicized species names.

5.	**Clarified sequencing depth normalization:** We have revised the description of the data normalization.

6.	**Updated references:** Duplicate references have been removed, and new references have been added. All reference numbers have been adjusted accordingly.

All changes are visible in the Revised Manuscript with Track Changes, and the line numbers in this response refer to this file. We hope that the revised manuscript meets your high standards. We also welcome any further constructive comments. Below, we provide point-by-point responses. Blue text indicates our responses, *italicized text* is from the manuscript, and red text highlights all modifications.

Best regards,

Mengnan He

**Response to Academic Editor**

**[Requirement 1]** Please ensure that your manuscript meets PLOS ONE's style requirements, including those for file naming. The PLOS ONE style templates can be found at <https://journals.plos.org/plosone/s/file?id=wjVg/PLOSOne_formatting_sample_main_body.pdf> and <https://journals.plos.org/plosone/s/file?id=ba62/PLOSOne_formatting_sample_title_authors_affiliations.pdf>

**Response:** We have carefully reviewed and adjusted our manuscript to meet PLOS ONE's style requirements, including file naming requirements. Please let us know if any further adjustments are needed.

**[Requirement 2]** Please note that PLOS One has specific guidelines on code sharing for submissions in which author-generated code underpins the findings in the manuscript. In these cases, we expect all author-generated code to be made available without restrictions upon publication of the work. Please review our guidelines at https://journals.plos.org/plosone/s/materials-and-software-sharing#loc-sharing-code and ensure that your code is shared in a way that follows best practice and facilitates reproducibility and reuse.

**Response:** We have reviewed PLOS ONE's guidelines on code sharing. We confirm that **no author-generated code** was produced or used in this study. All bioinformatics and statistical analyses were performed using publicly available software and standard R packages as detailed in the Methods section. Therefore, the code sharing requirement does not apply to this submission.

**[Requirement 3]** We note that the grant information you provided in the ‘Funding Information’ and ‘Financial Disclosure’ sections do not match.

When you resubmit, please ensure that you provide the correct grant numbers for the awards you received for your study in the ‘Funding Information’ section.

**Response:** Thanks for your kind reminders. We apologize for this oversight. We have reviewed our funding sources and prepared **an updated funding statement**, which has been included in the **cover letter** as requested. We confirm that the grant numbers for the awards we received for our study are correct in the ‘Funding Information’ section.

In addition, following the submission guidelines, we have removed the funding source that was inadvertently included in the Acknowledgments section and added the appropriate acknowledgements (Lines 416**–**421) in the Revised Manuscript with Track Changes.

**[Requirement 4]** If the reviewer comments include a recommendation to cite specific previously published works, please review and evaluate these publications to determine whether they are relevant and should be cited. There is no requirement to cite these works unless the editor has indicated otherwise.

**Response:** Thanks for your advice. Reviewer #1 recommended that the Discussion section would benefit from a more balanced comparison with previous microbiome studies in other captive wildlife species, but did not specify particular publications that must be cited.

The specific references we added are detailed in our response to Reviewer #1, Comment 9. We believe these changes have improved the balance and breadth of our Discussion section.

**[Requirement 5]** Please review your reference list to ensure that it is complete and correct. If you have cited papers that have been retracted, please include the rationale for doing so in the manuscript text, or remove these references and replace them with relevant current references. Any changes to the reference list should be mentioned in the rebuttal letter that accompanies your revised manuscript. If you need to cite a retracted article, indicate the article’s retracted status in the References list and also include a citation and full reference for the retraction notice.

**Response:** Thank you for this reminder. We have carefully reviewed our entire reference list and confirm that **no cited papers have been retracted**.

However, in response to Reviewer #1's suggestion that the Discussion section would benefit from a more balanced comparison with previous microbiome studies in other captive wildlife species, we have added **four new references** to the revised manuscript (new reference numbers **[53]**, **[54]**, **[56]**, and **[66]** as listed below). Consequently, all subsequent reference numbers have been adjusted accordingly.

Newly added references:

***[53]*** *Chen X, Chen W, Guo X, Zhang S, Xu B, Wu H, et al. Linking gut microbiota and stereotypic behavior of endangered species under ex situ conservation: first evidence from sun bears. Animals (Basel). 2025;15(3): 435. doi: 10.3390/ani15030435*

***[54]***	*Pan X, Liu F, Song Y, Wang H, Wang L, Qiu H, et al. Motor stereotypic behavior was associated with immune response in macaques: insight from transcriptome and gut microbiota analysis. Front Microbiol. 2021;12: 644540. doi: 10.3389/fmicb.2021.644540*

***[56]***	*Liu H, Xiao L, Liu Z, Deng Y, Zhu J, Yang C, et al. Impacts of captive domestication and geographical divergence on the gut microbiome of endangered forest musk deer. Animals (Basel). 2025;15(13): 1954. doi: 10.3390/ani15131954*

***[66]***	*Zhang X, Wang X, Ayala J, Liu Y, An J, Wang D, et al. Possible effects of early maternal separation on the gut microbiota of captive adult giant pandas. Animals (Basel). 2022;12(19): 2587. doi: 10.3390/ani12192587*

Additionally, we have removed **duplicate references** that were repeated in the original manuscript, and adjusted all reference numbers accordingly:

The original reference **[53]** (Wan et al., 2022) was **deleted** because it was a duplicate of reference **[20]**, which we retained.

Due to an inadvertent error, reference **[46]** (Kanehisa et al., 2017) was incorrectly cited at location **[64]**, resulting in duplicate citations. Therefore, the duplicate reference at **[64]** was **replaced** with a reference (Braga et al., 2024) that we originally intended to cite at [64], and this new reference was renumbered as **[67]**.

Due to an inadvertent error, reference **[47]** (Qin et al., 2012) was incorrectly cited at location **[65]**, resulting in duplicate citations. Therefore, the duplicate reference at **[65]** was **replaced** with a reference (Wang et al., 2025) that we originally intended to cite at [65], and this new reference was renumbered as **[68]**.

All changes are visible in the Revised Manuscript with Track Changes.

Newly added references:

***[67]*** *Braga JD, Thongngam M, Kumrungsee T. Gamma-aminobutyric acid as a potential postbiotic mediator in the gut-brain axis. NPJ Sci Food. 2024;8: 16. doi: 10.1038/s41538-024-00253-2*

***[68]*** *Wang D, Jiang Y, Jiang J, Pan Y, Yang Y, Fang X, et al. Gut microbial GABA imbalance emerges as a metabolic signature in mild autism spectrum disorder linked to overrepresented Escherichia. Cell Rep Med. 2025;6: 101919. doi: 10.1016/j.xcrm.2024.101919*

**Response to Reviewer #1**

**[Overall Evaluation]** This manuscript offers a compelling and innovative exploration of the link between gut microbiota and stereotypic behavior in captive giant pandas, employing both 16S rRNA sequencing and metagenomic analysis. The subject matter holds significance for the fields of animal behavior, conservation biology, and microbiome research.

**Response:** We sincerely thank you for your positive evaluation and constructive comments, which have helped us improve this manuscript. We have carefully addressed each point raised below.

**[Comment 1]** The study's inclusion of only 15 giant pandas, with subgroup analyses conducted on even smaller groups, significantly limits its statistical power and heightens the risk of false-positive associations.

The authors should critically address this limitation and justify how they can draw robust conclusions from such a small cohort.

**Response:** Thank you for raising this critical issue. We fully agree with this comment. Indeed, the small number of subjects (n=15) is a major limitation of our study. The giant panda is a rare species with a limited captive population, and individuals exhibiting obvious stereotypic behavior are even rarer, making it extremely difficult to find additional subjects. However, we would like to clarify that the limited number of individuals was a result of our rigorous selection from over 200 captive giant pandas at the Chengdu Giant Panda Breeding Research Base, rather than a random selection. The selection was based on behavioral observations during long-term captive husbandry, combined with short-term behavioral observations using a continuous recording method by trained researchers who had passed inter-observer and intra-observer reliability tests. Through this rigorous selection process, we only selected individuals with typical and clearly identifiable stereotypic behavior, ensuring the reliability of our results. We have added a more detailed explanation of the selection process to the Methods section (Lines 90**–**92 and 108**–**109). We acknowledge that this stringent selection resulted in a small number of individuals, which inevitably reduces statistical power. However, we prioritized data quality over quantity to ensure that our results reflect genuine biological differences.

Despite the limited number of individuals, we have taken multiple approaches to ensure the reliability of our findings:

**First**, although only 15 giant pandas were studied, we performed repeated sampling on each individual, which improved statistical power in mouse microbiota study (McAdams et al., 2026). Behavioral data of each giant panda were recorded over 10-day periods, and a total of 145 fecal samples were collected (approximately 10 feces per giant panda).

**Second**, to control for false-positive associations, we applied the **Benjamini–Hochberg correction** for multiple comparisons. This stringent statistical approach effectively reduces the risk of false-positive findings.

**Third**, to further validate our findings, we carefully compared our results with previously published studies. Our results are consistent with some earlier reports. For example, the significant difference in gut microbiota composition between the stereotypically behaving (SB) and normal groups has been reported in other captive wildlife species [53,54] and in human autism spectrum disorder studies [20,55]. This external consistency suggests that our observations reflect genuine biological differences.

**Moreover**, in the revised manuscript, we have further strengthened our acknowledgment of this limitation in the Discussion section, and justified how we mitigated the impact of this limitation to obtain more robust conclusions. In addition, we have explicitly defined this study as an **exploratory pilot study.** **All changes are visible in the Revised Manuscript with Track Changes.** **The above revisions are as follows (Lines 378–389)**:

*“The primary limitation of this study is the relatively small number of subjects, which inevitably limits the statistical power and heightens the risk of false-positive associations. However, this limitation stems from our rigorous selection, which ensured the reliability of our results. To mitigate concerns about this limitation, we performed repeated sampling on each individual (over 10-day behavioral observations and approximately 10 fecal samples per giant panda), which substantially improved statistical power. We also performed stratified analyses to control for confounding factors, and we applied stringent BH corrections for multiple comparisons. In addition to this primary limitation****,*** *the observed associations between specific microbes and stereotypic behavior are merely correlational, not causal. The underlying mechanisms by which these microbes may contribute to the development of stereotypic behavior remain speculative. Given these limitations, our research should be viewed as an exploratory pilot study. Therefore, studies with larger sample sizes and further experiments are needed to validate these preliminary findings.”*

We hope this response could address your concerns regarding this limitation.

Reference:

McAdams Z, Gustafson K, Ericsson A. Biological and technical variability in mouse microbiota analysis and implications for sample size determination. Lab Anim (NY). 2026;55(1): 29-34. doi: 10.1038/s41684-025-01664-8

**[Comment 2]** These variables may have a strong impact on gut microbiota composition and stereotypic behavior.

**Response:** Thank you for highlighting this important point. We fully agree that variables such as age, sex, diet, captive environment, and health status can profoundly influence both gut microbiota composition and stereotypic behavior.

In our study, we carefully controlled for these potential confounding factors. All selected giant pandas had comparable dietary structures, captive environments, and individual health statuses. **To clarify these controlled conditions, we have expanded our description in the Methods section (Lines 93–96) as follow:**

***“To examine the gut microbiome of the stereotypically behaving (SB) giant panda, fifteen captive giant pandas in the Chengdu Giant Panda Breeding Research Base were studied, all of which had comparable dietary structures (bamboo as the main food, supplemented with bamboo shoots, panda cake, and apple), feeding environments (each giant panda was housed in an independent enclosure with comparable indoor and outdoor space), and individual health statuses (all individuals confirmed to be healthy by routine veterinary examinations).”***

To further minimize the influence of age and sex, we performed stratified analyses (SB vs. normal geriatric females, and SB vs. normal adult males).

**[Comment 3]** While the reliability testing is commendable, greater methodological transparency is necessary.

**Response:** Thank you for acknowledging our reliability testing and the constructive suggestion regarding methodological transparency. In the original manuscript, we had already provided a detailed description of the reliability testing procedures in the Supporting Information (S2 File), as cited in the Methods section. However, we agree that the methodological transparency of the reliability testing should be strengthened. To address this concern, we have taken the following steps in the revised manuscript:

**First**, we have condensed the key methodological details of the reliability testing from S2 File and incorporated them into the Methods section (Lines 100–105) as follow:

“To ensure the reliability of observation, intra-observer and inter-observer reliability tests were conducted by two observers prior to formal observation. Intra-observer and inter-observer reliability were both assessed via Pearson correlation analysis on behavioral data obtained from seven 1-hour videos. For intra-observer reliability, data extracted by each observer twice at a one-week interval were compared; for inter-observer reliability, data extracted independently by the two observers were compared (see S2 File for a detailed description of measuring reliability).”

**Second**, we have added the results of reliability analysis to the main text (Lines 106–108) as follow:

“The reliability analysis passed, as the correlation coefficients (r) for both intra-observer (r = 0.98, S3 Table; r = 0.85, S4 Table) and inter-observer reliability (r = 0.95; S5 Table) exceeded 70% [22].”

**Third**, we have further expanded the description of the reliability analysis samples in Supporting Information (S2 File, Lines 16–17):

*“The seven 1-hour videos were recorded from different stereotypically behaving giant pandas respectively.”*

We believe these revisions substantially enhance the methodological transparency of the reliability testing.

**[Comment 4]** The manuscript contains several grammatical and typographical errors that require thorough English language editing.

**Response:** Thank you for this suggestion. Following a professional language edit, we have further carefully re-examined the entire manuscript and have made comprehensive corrections to address grammatical and typographical issues. All changes are visible in the Revised Manuscript with Track Changes.

**[Comment 5]** Species names should be consistently italicized throughout the manuscript.

**Response:** Thank you for this reminder. We have fully checked and revised the entire manuscript, including Fig 2, to ensure that all species names are consistently italicized. All changes are visible in the Revised Manuscript with Track Changes.

**[Comment 6]** The abbreviation “SB” should be clearly defined at its first appearance in each major section.

**Response:** Thank you for this suggestion. We have defined “SB” at its first appearance in each major section (Abstract, Introduction, Materials and methods, Results, Discussion, and Conclusion) by writing “stereotypically behaving (SB)” in the revised manuscript.

**[Comment 7]** Figure legends should be simplified and made more concise for better readability.

**Response:** Thank you for this valuable suggestion. We have revised the figure legends for Figs 1–5 in the main text, as well as S1 Fig and S3 Fig in the Supporting Information, to make them more simplified and concise. All changes are visible in the Revised Manuscript with Track Changes.

**[Comment 8]** Please clarify whether sequencing depth normalization was performed prior to all diversity analyses.

**Response:** Thank you for this question. **Yes, sequencing depth normalization was performed prior to all diversity analyses**. We have revised the Methods section to clarify this procedure as follows:

**First**, we have added a clear definition of ASVs (Lines 135–136):

*“The effective tags were subsequently denoised using DADA2 within the QIIME2 software (Version QIIME2-202202), each de‐duplicated sequence generated after noise reduction using DADA2 is called Amplicon Sequence Variant (ASV).”*

**Second**, we have revised the description of the data normalization (Lines 140–142) to more explicitly state that sequencing depth normalization was performed:

*“Additionally, to correct biases caused by sequencing depth, the absolute abundance of ASVs was normalized using a standard of sequence number corresponding to the sample with the least sequences.”*

**[Comment 9]** The Discussion section would benefit from a more balanced comparison with previous microbiome studies in other captive wildlife species.

**Response:** Thank you for this constructive suggestion. In the revised manuscript, we have expanded the Discussion section to include more balanced comparisons with previous microbiome studies in other captive wildlife species. Specifically, we have added the following content:

**Added to Lines 325–326:**

“Similar alterations have also been observed in other captive wild animals displaying stereotypic behavior [53,54].”

These references report that both captive sun bears [53] and captive macaques [54] exhibiting stereotypic behavior showed significant gut microbiota alterations compared with that in their normal controls, which is consistent with our findings in giant pandas.

**Added to Lines 335–338:**

“Similarly, significantly higher Clostridium abundance has been observed in captive forest musk deer than in wild populations [56], suggesting that gut microbial alterations may be associated with environmental stress in a captive environment.”

Liu et al. [56] reported abnormal proliferation of Clostridium in captive forest musk deer compared to wild controls, suggesting that the enrichment of Clostridium in SB giant pandas may be linked to captive environment, where stereotypic behavior is prevalent.

**Added to Lines 359–363:**

“It has been reported that maternal deprivation during the early life of captive giant pandas, a key determinant of abnormal behavior in stress model individuals, increased the relative abundance of Clostridium bacteria, which is significantly and positively correlated with propionic acid metabolism [66].”

This research [66], which also studied captive giant pandas, provides support for our hypothesis that enhanced abundance of *Clostridium* bacteria in the gut microbiome, accompanied by elevated levels of propionate, may be associated with the emergence of stereotypic behavior in giant pandas through the gut-brain axis.

Newly added references:

***[53]*** *Chen X, Chen W, Guo X, Zhang S, Xu B, Wu H, et al. Linking gut microbiota and stereotypic behavior of endangered species under ex situ conservation: first evidence from sun bears. Animals (Basel). 2025;15(3): 435. doi: 10.3390/ani15030435*

***[54]***	*Pan X, Liu F, Song Y, Wang H, Wang L, Qiu H, et al. Motor stereotypic behavior was associated with immune response in macaques: insight from transcriptome and gut microbiota analysis. Front Microbiol. 2021;12: 644540. doi: 10.3389/fmicb.2021.644540*

***[56]***	*Liu H, Xiao L, Liu Z, Deng Y, Zhu J, Yang C, et al. Impacts of captive domestication and geographical divergence on the gut microbiome of endangered forest musk deer. Animals (Basel). 2025;15(13): 1954. doi: 10.3390/ani15131954*

***[66]***	*Zhang X, Wang X, Ayala J, Liu Y, An J, Wang D, et al. Possible effects of early maternal separation on the gut microbiota of captive adult giant pandas. Animals (Basel). 2022;12(19): 2587. doi: 10.3390/ani12192587*

**[Comment 10]** Several references appear conceptually duplicated and could be reduced for conciseness. For instance, KEGG references are repeated multiple times.

**Response:** Thank you for pointing out this issue. we have removed **duplicate references** that were repeated in the original manuscript. Because we have also added several new references to the Discussion section in response to your suggestion for a more balanced comparison with previous microbiome studies in other captive wildlife species, all reference numbers have been renumbered accordingly:

The original reference **[53]** (Wan et al., 2022) was **deleted** because it was a duplicate of reference **[20]**, which we retained.

Due to an inadvertent error, reference **[46]** (Kanehisa et al., 2017) was incorrectly cited at location **[64]**, resulting in duplicate citations. Therefore, the duplicate reference at **[64]** was **replaced** with a reference (Braga et al., 2024) that we originally intended to cite at [64], and this new reference was renumbered as **[67]**.

Due to an inadvertent error, reference **[47]** (Qin et al., 2012) was incorrectly cited at location **[65]**, resulting in duplicate citations. Therefore, the duplicate reference at **[65]** was **replaced** with a reference (Wang et al., 2025) that we originally intended to cite at [65], and this new reference was renumbered as **[68]**.

These changes are visible in the Revised Manuscript with Track Changes.

Newly added references:

***[67]*** *Braga JD, Thongngam M, Kumrungsee T. Gamma-aminobutyric acid as a potential postbiotic mediator in the gut-brain axis. NPJ Sci Food. 2024;8: 16. doi: 10.1038/s41538-024-00253-2*

***[68]*** *Wang D, Jiang Y, Jiang J, Pan Y, Yang Y, Fang X, et al. Gut microbial GABA imbalance emerges as a metabolic signature in mild autism spectrum disorder linked to overrepresented Escherichia. Cell Rep Med. 2025;6: 101919. doi: 10.1016/j.xcrm.2024.101919*

**Response to Reviewer #2**

**[General Comment]** This manuscript investigates the association between gut microbiota and stereotypic behavior in captive giant pandas through behavioral observations, 16S rRNA gene sequencing, and metagenomic analyses. The study addresses an important conservation and animal welfare issue and provides novel evidence suggesting that gut microbial dysbiosis may be associated with stereotypic behavior in giant pandas. The integration of behavioral observations with microbiome and functional metagenomic analyses is a notable strength. The identification of potential microbial biomarkers, particularly *Clostridium* spp. and *Serratia* sp. *Se-RSBMAAmG*, contributes valuable preliminary data to the field.

**Response:** Thank you for the positive comments on our manuscript. We appreciate the acknowledgement that our study addresses an important conservation and animal welfare issue and that the integration of behavioral observations with microbiome and functional metagenomic analyses is a notable strength. We also appreciate the acknowledgment that our identification of potential microbial biomarkers, particularly *Clostridium* spp. and *Serratia* sp. *Se-BMAAmG*. This acknowledgment highlights key directions for future research.

We have carefully examined and verified the entire manuscript. We have also worked on both language and readability and made every effort to address any potential issues in our manuscript. In addition, we have carefully addressed all comments raised by Reviewer #1 and the academic editor, and all revisions are visible in the **Revised Manuscript with Track Changes**. We believe these revisions have further strengthened the quality of our work.

**Additional Corrections**

We have corrected the cell format in **column H** (the 8th column) of **S2–S5 Tables**, where the cells were inadvertently set to percentage format. The cells have now been adjusted to the **general number format**. The raw numerical values remain unchanged, and **none of our conclusions are affected**.
