# Supplementary material for: Gut microbiome is associated with stereotypic behavior of the giant panda (Ailuropoda melanoleuca)
Source: PLoS One. 2026 Sep 23;21(9):e0357625. doi: 10.1371/journal.pone.0357625 (PMC13600500; doi:10.1371/journal.pone.0357625)
Supplement: Peer review history

# Peer review: Decision Letter 0

**DOI:** 10.1371/journal.pone.0357625.r001

**Authors:** Sayed Haidar Abbas Raza

17 Jun 2026

Dear Dr. He,

Please submit your revised manuscript by Aug 01 2026 11:59PM. If you will need more time than this to complete your revisions, please reply to this message or contact the journal office at plosone@plos.org. When you're ready to submit your revision, log on to https://www.editorialmanager.com/pone/ and select the 'Submissions Needing Revision' folder to locate your manuscript file.

- A letter that responds to each point raised by the academic editor and reviewer(s). You should upload this letter as a separate file labeled 'Response to Reviewers'.
- A marked-up copy of your manuscript that highlights changes made to the original version. You should upload this as a separate file labeled 'Revised Manuscript with Track Changes'.
- An unmarked version of your revised paper without tracked changes. You should upload this as a separate file labeled 'Manuscript'.

If you would like to make changes to your financial disclosure, please include your updated statement in your cover letter. Guidelines for resubmitting your figure files are available below the reviewer comments at the end of this letter.

If applicable, we recommend that you deposit your laboratory protocols in protocols.io to enhance the reproducibility of your results. Protocols.io assigns your protocol its own identifier (DOI) so that it can be cited independently in the future. For instructions see: https://journals.plos.org/plosone/s/submission-guidelines#loc-laboratory-protocols. Additionally, PLOS ONE offers an option for publishing peer-reviewed Lab Protocol articles, which describe protocols hosted on protocols.io. Read more information on sharing protocols at https://plos.org/protocols?utm_medium=editorial-email&utm_source=authorletters&utm_campaign=protocols.

As the corresponding author, your ORCID iD is verified in the submission system and will appear in the published article. PLOS supports the use of ORCID, and we encourage all coauthors to register for an ORCID iD and use it as well. Please encourage your coauthors to verify their ORCID iD within the submission system before final acceptance, as unverified ORCID iDs will not appear in the published article. *Only*  the individual author can complete the verification step; PLOS staff *cannot*  verify ORCID iDs on behalf of authors.

We look forward to receiving your revised manuscript.

Kind regards,

Sayed Haidar Abbas Raza, PhD

Academic Editor

PLOS One

Journal Requirements:

When submitting your revision, we need you to address these additional requirements.

1. Please ensure that your manuscript meets PLOS ONE's style requirements, including those for file naming. The PLOS ONE style templates can be found at

https://journals.plos.org/plosone/s/file?id=wjVg/PLOSOne_formatting_sample_main_body.pdf and

https://journals.plos.org/plosone/s/file?id=ba62/PLOSOne_formatting_sample_title_authors_affiliations.pdf

2. Please note that PLOS One has specific guidelines on code sharing for submissions in which author-generated code underpins the findings in the manuscript. In these cases, we expect all author-generated code to be made available without restrictions upon publication of the work. Please review our guidelines at https://journals.plos.org/plosone/s/materials-and-software-sharing#loc-sharing-code and ensure that your code is shared in a way that follows best practice and facilitates reproducibility and reuse.

3. We note that the grant information you provided in the ‘Funding Information’ and ‘Financial Disclosure’ sections do not match.

When you resubmit, please ensure that you provide the correct grant numbers for the awards you received for your study in the ‘Funding Information’ section.

4. If the reviewer comments include a recommendation to cite specific previously published works, please review and evaluate these publications to determine whether they are relevant and should be cited. There is no requirement to cite these works unless the editor has indicated otherwise.

5. Please review your reference list to ensure that it is complete and correct. If you have cited papers that have been retracted, please include the rationale for doing so in the manuscript text, or remove these references and replace them with relevant current references. Any changes to the reference list should be mentioned in the rebuttal letter that accompanies your revised manuscript. If you need to cite a retracted article, indicate the article’s retracted status in the References list and also include a citation and full reference for the retraction notice.

[Note: HTML markup is below. Please do not edit.]

Reviewers' comments:

Reviewer's Responses to Questions

**Comments to the Author**

1. Is the manuscript technically sound, and do the data support the conclusions?

Reviewer #1: Yes

Reviewer #2: Yes

**********

2. Has the statistical analysis been performed appropriately and rigorously?

Reviewer #1: Yes

Reviewer #2: Yes

**********

3. Have the authors made all data underlying the findings in their manuscript fully available?

The PLOS Data policy

Reviewer #1: Yes

Reviewer #2: Yes

**********

4. Is the manuscript presented in an intelligible fashion and written in standard English?

Reviewer #1: Yes

Reviewer #2: Yes

**********

Reviewer #1: Title: Gut microbiome is associated with stereotypic behavior of the giant panda (Ailuropoda melanoleuca)

Overall Evaluation

This manuscript offers a compelling and innovative exploration of the link between gut microbiota and stereotypic behavior in captive giant pandas, employing both 16S rRNA sequencing and metagenomic analysis. The subject matter holds significance for the fields of animal behavior, conservation biology, and microbiome research.

Comments

�The study's inclusion of only 15 giant pandas, with subgroup analyses conducted on even smaller groups, significantly limits its statistical power and heightens the risk of false-positive associations.

�The authors should critically address this limitation and justify how they can draw robust conclusions from such a small cohort.

�These variables may have a strong impact on gut microbiota composition and stereotypic behavior.

�While the reliability testing is commendable, greater methodological transparency is necessary.

�The manuscript contains several grammatical and typographical errors that require thorough English language editing.

�Species names should be consistently italicized throughout the manuscript.

�The abbreviation “SB” should be clearly defined at its first appearance in each major section.

�Figure legends should be simplified and made more concise for better readability.

�Please clarify whether sequencing depth normalization was performed prior to all diversity analyses.

�The Discussion section would benefit from a more balanced comparison with previous microbiome studies in other captive wildlife species.

�Several references appear conceptually duplicated and could be reduced for conciseness. For instance, KEGG references are repeated multiple times.

Recommendation

Accept

The study presents novel and potentially valuable insights, yet minor revisions are necessary to enhance its quality before it is ready for publication.

Reviewer #2: This manuscript investigates the association between gut microbiota and stereotypic behavior in captive giant pandas through behavioral observations, 16S rRNA gene sequencing, and metagenomic analyses. The study addresses an important conservation and animal welfare issue and provides novel evidence suggesting that gut microbial dysbiosis may be associated with stereotypic behavior in giant pandas. The integration of behavioral observations with microbiome and functional metagenomic analyses is a notable strength. The identification of potential microbial biomarkers, particularly Clostridium spp. and Serratia sp. Se-RSBMAAmG, contributes valuable preliminary data to the field.

**********

what does this mean?). If published, this will include your full peer review and any attached files.

If you choose “no”, your identity will remain anonymous but your review may still be made public.

**Do you want your identity to be public for this peer review?**  For information about this choice, including consent withdrawal, please see our Privacy Policy

Reviewer #1: **Yes:** RAMYA SRI SURA

Reviewer #2: **Yes:** Sai Kumar Badam

**********

[NOTE: If reviewer comments were submitted as an attachment file, they will be attached to this email and accessible via the submission site. Please log into your account, locate the manuscript record, and check for the action link "View Attachments". If this link does not appear, there are no attachment files.]

To ensure your figures meet our technical requirements, please review our figure guidelines: https://journals.plos.org/plosone/s/figures

You may also use PLOS’s free figure tool, NAAS, to help you prepare publication quality figures: https://journals.plos.org/plosone/s/figures#loc-tools-for-figure-preparation.

NAAS will assess whether your figures meet our technical requirements by comparing each figure against our figure specifications.

# Author comment: Author response to Decision Letter 1

**DOI:** 10.1371/journal.pone.0357625.r002

31 Jul 2026

Response to Editor and Reviewers

Dear Editor and Reviewers,

We genuinely appreciate you for taking the time to review our manuscript and provide valuable comments. Your insightful comments have significantly contributed to the improvements in the current version. We have carefully considered each comment and have made every effort to address them. The following is a summary of the major revisions in the revised manuscript:

1. Response to concerns regarding the sample size: We have acknowledged the small sample size as a limitation, clarified that this limitation stems from our rigorous selection of subjects, and supported the robustness of our conclusions by highlighting our sampling strategy, application of Benjamini–Hochberg correction, and consistency with previous studies. We have also explicitly defined this study as an exploratory pilot study in the revised Discussion section.

2. Improved methodological transparency: We have expanded the description of controlled variables and incorporated condensed reliability testing procedures from the Supporting Information into the Methods section.

3. Expanded Discussion with cross-species comparisons: We have added new references and discussions comparing our findings with microbiome studies in other captive wildlife species.

4. Improved readability: We have simplified the figure legends, corrected grammatical and typographical errors, clearly defined the abbreviation "SB", and italicized species names.

5. Clarified sequencing depth normalization: We have revised the description of the data normalization.

6. Updated references: Duplicate references have been removed, and new references have been added. All reference numbers have been adjusted accordingly.

All changes are visible in the Revised Manuscript with Track Changes, and the line numbers in this response refer to this file. We hope that the revised manuscript meets your high standards. We also welcome any further constructive comments. Below, we provide point-by-point responses. Blue text indicates our responses, italicized text is from the manuscript, and red text highlights all modifications.

Best regards,

Mengnan He

Response to Academic Editor

[Requirement 1] Please ensure that your manuscript meets PLOS ONE's style requirements, including those for file naming. The PLOS ONE style templates can be found at https://journals.plos.org/plosone/s/file?id=wjVg/PLOSOne_formatting_sample_main_body.pdf and https://journals.plos.org/plosone/s/file?id=ba62/PLOSOne_formatting_sample_title_authors_affiliations.pdf

Response: We have carefully reviewed and adjusted our manuscript to meet PLOS ONE's style requirements, including file naming requirements. Please let us know if any further adjustments are needed.

[Requirement 2] Please note that PLOS One has specific guidelines on code sharing for submissions in which author-generated code underpins the findings in the manuscript. In these cases, we expect all author-generated code to be made available without restrictions upon publication of the work. Please review our guidelines at https://journals.plos.org/plosone/s/materials-and-software-sharing#loc-sharing-code and ensure that your code is shared in a way that follows best practice and facilitates reproducibility and reuse.

Response: We have reviewed PLOS ONE's guidelines on code sharing. We confirm that no author-generated code was produced or used in this study. All bioinformatics and statistical analyses were performed using publicly available software and standard R packages as detailed in the Methods section. Therefore, the code sharing requirement does not apply to this submission.

[Requirement 3] We note that the grant information you provided in the ‘Funding Information’ and ‘Financial Disclosure’ sections do not match.

When you resubmit, please ensure that you provide the correct grant numbers for the awards you received for your study in the ‘Funding Information’ section.

Response: Thanks for your kind reminders. We apologize for this oversight. We have reviewed our funding sources and prepared an updated funding statement, which has been included in the cover letter as requested. We confirm that the grant numbers for the awards we received for our study are correct in the ‘Funding Information’ section.

In addition, following the submission guidelines, we have removed the funding source that was inadvertently included in the Acknowledgments section and added the appropriate acknowledgements (Lines 416–421) in the Revised Manuscript with Track Changes.

[Requirement 4] If the reviewer comments include a recommendation to cite specific previously published works, please review and evaluate these publications to determine whether they are relevant and should be cited. There is no requirement to cite these works unless the editor has indicated otherwise.

Response: Thanks for your advice. Reviewer #1 recommended that the Discussion section would benefit from a more balanced comparison with previous microbiome studies in other captive wildlife species, but did not specify particular publications that must be cited.

The specific references we added are detailed in our response to Reviewer #1, Comment 9. We believe these changes have improved the balance and breadth of our Discussion section.

[Requirement 5] Please review your reference list to ensure that it is complete and correct. If you have cited papers that have been retracted, please include the rationale for doing so in the manuscript text, or remove these references and replace them with relevant current references. Any changes to the reference list should be mentioned in the rebuttal letter that accompanies your revised manuscript. If you need to cite a retracted article, indicate the article’s retracted status in the References list and also include a citation and full reference for the retraction notice.

Response: Thank you for this reminder. We have carefully reviewed our entire reference list and confirm that no cited papers have been retracted.

However, in response to Reviewer #1's suggestion that the Discussion section would benefit from a more balanced comparison with previous microbiome studies in other captive wildlife species, we have added four new references to the revised manuscript (new reference numbers [53], [54], [56], and [66] as listed below). Consequently, all subsequent reference numbers have been adjusted accordingly.

Newly added references:

[53] Chen X, Chen W, Guo X, Zhang S, Xu B, Wu H, et al. Linking gut microbiota and stereotypic behavior of endangered species under ex situ conservation: first evidence from sun bears. Animals (Basel). 2025;15(3): 435. doi: 10.3390/ani15030435

[54] Pan X, Liu F, Song Y, Wang H, Wang L, Qiu H, et al. Motor stereotypic behavior was associated with immune response in macaques: insight from transcriptome and gut microbiota analysis. Front Microbiol. 2021;12: 644540. doi: 10.3389/fmicb.2021.644540

[56] Liu H, Xiao L, Liu Z, Deng Y, Zhu J, Yang C, et al. Impacts of captive domestication and geographical divergence on the gut microbiome of endangered forest musk deer. Animals (Basel). 2025;15(13): 1954. doi: 10.3390/ani15131954

[66] Zhang X, Wang X, Ayala J, Liu Y, An J, Wang D, et al. Possible effects of early maternal separation on the gut microbiota of captive adult giant pandas. Animals (Basel). 2022;12(19): 2587. doi: 10.3390/ani12192587

Additionally, we have removed duplicate references that were repeated in the original manuscript, and adjusted all reference numbers accordingly:

The original reference [53] (Wan et al., 2022) was deleted because it was a duplicate of reference [20], which we retained.

Due to an inadvertent error, reference [46] (Kanehisa et al., 2017) was incorrectly cited at location [64], resulting in duplicate citations. Therefore, the duplicate reference at [64] was replaced with a reference (Braga et al., 2024) that we originally intended to cite at [64], and this new reference was renumbered as [67].

Due to an inadvertent error, reference [47] (Qin et al., 2012) was incorrectly cited at location [65], resulting in duplicate citations. Therefore, the duplicate reference at [65] was replaced with a reference (Wang et al., 2025) that we originally intended to cite at [65], and this new reference was renumbered as [68].

All changes are visible in the Revised Manuscript with Track Changes.

Newly added references:

[67] Braga JD, Thongngam M, Kumrungsee T. Gamma-aminobutyric acid as a potential postbiotic mediator in the gut-brain axis. NPJ Sci Food. 2024;8: 16. doi: 10.1038/s41538-024-00253-2

[68] Wang D, Jiang Y, Jiang J, Pan Y, Yang Y, Fang X, et al. Gut microbial GABA imbalance emerges as a metabolic signature in mild autism spectrum disorder linked to overrepresented Escherichia. Cell Rep Med. 2025;6: 101919. doi: 10.1016/j.xcrm.2024.101919

Response to Reviewer #1

[Overall Evaluation] This manuscript offers a compelling and innovative exploration of the link between gut microbiota and stereotypic behavior in captive giant pandas, employing both 16S rRNA sequencing and metagenomic analysis. The subject matter holds significance for the fields of animal behavior, conservation biology, and microbiome research.

Response: We sincerely thank you for your positive evaluation and constructive comments, which have helped us improve this manuscript. We have carefully addressed each point raised below.

[Comment 1] The study's inclusion of only 15 giant pandas, with subgroup analyses conducted on even smaller groups, significantly limits its statistical power and heightens the risk of false-positive associations.

The authors should critically address this limitation and justify how they can draw robust conclusions from such a small cohort.

Response: Thank you for raising this critical issue. We fully agree with this comment. Indeed, the small number of subjects (n=15) is a major limitation of our study. The giant panda is a rare species with a limited captive population, and individuals exhibiting obvious stereotypic behavior are even rarer, making it extremely difficult to find additional subjects. However, we would like to clarify that the limited number of individuals was a result of our rigorous selection from over 200 captive giant pandas at the Chengdu Giant Panda Breeding Research Base, rather than a random selection. The selection was based on behavioral observations during long-term captive husbandry, combined with short-term behavioral observations using a continuous recording method by trained researchers who had passed inter-observer and intra-observer reliability tests. Through this rigorous selection process, we only selected individuals with typical and clearly identifiable stereotypic behavior, ensuring the reliability of our results. We have added a more detailed explanation of the selection process to the Methods section (Lines 90–92 and 108–109). We acknowledge that this stringent selection resulted in a small number of individuals, which inevitably reduces statistical power. However, we prioritized data quality over quantity to ensure that our results reflect genuine biological differences.

Despite the limited number of individuals, we have taken multiple approaches to ensure the reliability of our findings:

First, although only 15 giant pandas were studied, we performed repeated sampling on each individual, which improved statistical power in mouse microbiota study (McAdams et al., 2026). Behavioral data of each giant panda were recorded over 10-day periods, and a total of 145 fecal samples were collected (approximately 10 feces per giant panda).

Second, to control for false-positive associations, we applied the Benjamini–Hochberg correction for multiple comparisons. This stringent statistical approach effectively reduces the risk of false-positive findings.

Third, to further validate our findings, we carefully compared our results with previously published studies. Our results are consistent with some earlier reports. For example, the significant difference in gut microbiota composition between the stereotypically behaving (SB) and normal groups has been reported in other captive wildlife species [53,54] and in human autism spectrum disorder studies [20,55]. This external consistency suggests that our observations reflect genuine biological differences.

Moreover, in the revised manuscript, we have further strengthened our acknowledgment of this limitation in the Discussion section, and justified how we mitigated the impact of this limitation to obtain more robust conclusions. In addition, we have explicitly defined this study as an exploratory pilot study. All changes are visible in the Revised Manuscript with Track Changes. The above revisions are as follows (Lines 378–389):

“The primary limitation of this study is the relatively small number of subjects, which inevitably limits the statistical power and heightens the risk of false-positive associations. However, this limitation stems from our rigorous selection, which ensured the reliability of our results. To mitigate concerns about this limitation, we performed repeated sampling on each individual (over 10-day behavioral observations and approximately 10 fecal samples per giant panda), which substantially improved statistical power. We also performed stratified analyses to control for confounding factors, and we applied stringent BH corrections for multiple comparisons. In addition to this primary limitation, the observed associations between specific microbes and stereotypic behavior are merely correlational, not causal. The underlying mechanisms by which these microbes may contribute to the development of stereotypic behavior remain speculative. Given these limitations, our research should be viewed as an exploratory pilot study. Therefore, studies with larger sample sizes and further experiments are needed to validate these preliminary findings.”

We hope this response could address your concerns regarding this limitation.

Reference:

McAdams Z, Gustafson K, Ericsson A. Biological and technical variability in mouse microbiota analysis and implications for sample size determination. Lab Anim (NY). 2026;55(1): 29-34. doi: 10.1038/s41684-025-01664-8

[Comment 2] These variables may have a strong impact on gut microbiota composition and stereotypic behavior.

Response: Thank you for highlighting this important point. We fully agree that variables such as age, sex, diet, captive environment, and health status can profoundly influence both gut microbiota composition and stereotypic behavior.

In our study, we carefully controlled for these potential confounding factors. All selected giant pandas had comparable dietary structures, captive environments, and individual health statuses. To clarify these controlled conditions, we have expanded our description in the Methods section (Lines 93–96) as follow:

“To examine the gut microbiome of the stereotypically behaving (SB) giant panda, fifteen captive giant pandas in the Chengdu Giant Panda Breeding Research Base were studied, all of which had comparable dietary structures (bamboo as the main food, supplemented with bamboo shoots, panda cake, and apple), feeding environments (each giant panda was housed in an independent enclosure with comparable indoor and outdoor space), and individual health statuses (all individuals confirmed to be healthy by routine veterinary examinations).”

To further minimize the influence of age and sex, we performed stratified analyses (SB vs. normal geriatric females, and SB vs. normal adult males).

[Comment 3] While the reliability testing is commendable, greater methodological transparency is necessary.

Response: Thank you for acknowledging our reliability testing and the constructive suggestion regarding methodological transparency. In the original manuscript, we had already provided a detailed description of the reliability testing procedures in the Supporting Information (S2 File), as cited in the Methods section. However, we agree that the methodological transparency of the reliability testing should be strengthened. To address this concern, we have taken the following steps in the revised manuscript:

First, we have condensed the key methodological details of the reliability testin

Attachment Submitted filename: Response to Reviewers.doc

# Peer review: Decision Letter 1

**DOI:** 10.1371/journal.pone.0357625.r003

**Authors:** Sayed Haidar Abbas Raza

19 Aug 2026

Gut microbiome is associated with stereotypic behavior of the giant panda (Ailuropoda melanoleuca)

PONE-D-26-21927R1

Dear Dr. He,

We’re pleased to inform you that your manuscript has been judged scientifically suitable for publication and will be formally accepted for publication once it meets all outstanding technical requirements.

Within one week, you’ll receive an e-mail detailing the required amendments. When these have been addressed, you’ll receive a formal acceptance letter and your manuscript will be scheduled for publication.

An invoice will be generated when your article is formally accepted. Please note, if your institution has a publishing partnership with PLOS and your article meets the relevant criteria, all or part of your publication costs will be covered. Please make sure your user information is up-to-date by logging into Editorial Manager at Editorial Manager® and clicking the ‘Update My Information' link at the top of the page. For questions related to billing, please contact billing support.

If your institution or institutions have a press office, please notify them about your upcoming paper to help maximize its impact. If they’ll be preparing press materials, please inform our press team as soon as possible -- no later than 48 hours after receiving the formal acceptance. Your manuscript will remain under strict press embargo until 2 pm Eastern Time on the date of publication. For more information, please contact onepress@plos.org.

Kind regards,

Sayed Haidar Abbas Raza, PhD

Academic Editor

PLOS One

Additional Editor Comments (optional):

Reviewers' comments:

Reviewer's Responses to Questions

**Comments to the Author**

Reviewer #2: All comments have been addressed

**********

2. Is the manuscript technically sound, and do the data support the conclusions?

Reviewer #2: Yes

**********

3. Has the statistical analysis been performed appropriately and rigorously?

Reviewer #2: Yes

**********

4. Have the authors made all data underlying the findings in their manuscript fully available?

The PLOS Data policy

Reviewer #2: Yes

**********

5. Is the manuscript presented in an intelligible fashion and written in standard English?

Reviewer #2: Yes

**********

Reviewer #2: The study addresses an important conservation and animal welfare issue and provides novel evidence suggesting that gut microbial dysbiosis may be associated with stereotypic behavior in giant pandas. The integration of behavioral observations with microbiome and functional metagenomic analyses is a notable strength. The identification of potential microbial biomarkers, particularly Clostridium spp. and Serratia sp. Se-RSBMAAmG, contributes valuable preliminary data to the field.

**********

what does this mean?). If published, this will include your full peer review and any attached files.

If you choose “no”, your identity will remain anonymous but your review may still be made public.

**Do you want your identity to be public for this peer review?**  For information about this choice, including consent withdrawal, please see our Privacy Policy

Reviewer #2: **Yes:** Sai Kumar Badam

**********

# Editor report: Acceptance letter

**DOI:** 10.1371/journal.pone.0357625.r004

**Authors:** Sayed Haidar Abbas Raza

PONE-D-26-21927R1

PLOS One

Dear Dr. He,

I'm pleased to inform you that your manuscript has been deemed suitable for publication in PLOS One. Congratulations! Your manuscript is now being handed over to our production team.

At this stage, our production department will prepare your paper for publication. This includes ensuring the following:

* All references, tables, and figures are properly cited

* All relevant supporting information is included in the manuscript submission,

* There are no issues that prevent the paper from being properly typeset

You will receive further instructions from the production team, including instructions on how to review your proof when it is ready. Please keep in mind that we are working through a large volume of accepted articles, so please give us a few days to review your paper and let you know the next and final steps.

Lastly, if your institution or institutions have a press office, please let them know about your upcoming paper now to help maximize its impact. If they'll be preparing press materials, please inform our press team within the next 48 hours. Your manuscript will remain under strict press embargo until 2 pm Eastern Time on the date of publication. For more information, please contact onepress@plos.org.

You will receive an invoice from PLOS for your publication fee after your manuscript has reached the completed accept phase. If you receive an email requesting payment before acceptance or for any other service, this may be a phishing scheme. Learn how to identify phishing emails and protect your accounts at https://explore.plos.org/phishing.

If we can help with anything else, please email us at customercare@plos.org.

Thank you for submitting your work to PLOS One and supporting open access.

Kind regards,

PLOS One Editorial Office Staff

on behalf of

Dr. Sayed Haidar Abbas Raza

Academic Editor

PLOS One
